# Pharmacokinetics of cannabidiol, (-)-*trans*-Δ^9^-tetrahydrocannabinol, and their oxidative metabolites after intravenous and oral administration of a cannabidiol-dominant full-spectrum hemp product to beagle dogs

**DOI:** 10.3389/fvets.2025.1556975

**Published:** 2025-04-08

**Authors:** Susanna E. Kitts-Morgan, Richard A. Sams, William W. Muir

**Affiliations:** ^1^Physiology, College of Veterinary Medicine, Lincoln Memorial University, Harrogate, TN, United States; ^2^KCA Laboratories, Nicholasville, KY, United States

**Keywords:** dog, cannabidiol, pharmacokinetics, adverse effects, oral dosing, intravenous dosing

## Abstract

**Introduction:**

This study investigated the pharmacokinetics, safety, and tolerability of a full-spectrum CBD-dominant oil formulated in medium-chain triglycerides (MCT oil) after a single intravenous (IV) administration, a single oral (PO) administration, and multiple oral administrations of CBD at a dose of 2.2 mg/kg in adult male and female beagle dogs.

**Methods:**

The CBD-dominant extract was administered to adult, intact beagle dogs (male *n* = 4, female *n* = 2) once intravenously, once orally, and every 12 h orally for 21 days at a dose of 2.2 mg CBD/kg body weight (BW). Blood samples were collected at predetermined times to measure concentrations of serum CBD, 7-hydroxy-CBD (7-OH-CBD), 7-*nor*-7-carboxy-CBD (7-COOH-CBD), Δ^9^-tetrahydrocannabinol (Δ^9^-THC), 11-hydroxy-THC (11-OH-THC), and 11-carboxy-THC (11-COOH-THC). Serum CBD and Δ^9^-THC concentrations were analyzed to estimate various pharmacokinetic parameters. Selected physical, behavioral, hematologic, and blood chemical measurements were obtained before and during single and repeated dose administrations.

**Results:**

Pharmacokinetics of CBD after IV administration indicated a median (range) systemic clearance (CL) of 7.06 (6.14–10.5) mL/min/kg, a steady-state volume of distribution (V_ss_) of 2.13 (1.10–2.85) L/kg, and a half-life of 291 (183–508) min. The median (range) extent of systemic availability of CBD after a single oral dose was 31.2 (17.7–35.7)%. Pharmacokinetics of Δ^9^-THC after IV administration were characterized by a CL of 8.85 (6.88–14.4) mL/min/kg, V_ss_ of 1.98 (1.30–2.30) L/kg, and a half-life of 169 (139–476) min. The extent of systemic availability of Δ^9^-THC after PO administration was 40.9 (20.5–46.2)%. The test article was well tolerated in all dogs during the study. Although serum alkaline phosphatase concentrations increased during the repeated PO dose study, they remained within normal limits.

**Discussion:**

Both CBD and Δ^9^-THC were rapidly cleared after IV administration and exhibited extensive volumes of distribution. Comparison of clearance to serum hepatic blood flow estimated the hepatic extraction ratio and extent of first pass metabolism after PO administration, which was confirmed by analyzing the single PO dose pharmacokinetic data. The AUC_0−∞_ for 7-OH-CBD after single IV compared to single PO dose was not different, suggesting complete absorption of CBD from the formulation in MCT oil when administered with canned dog food.

## 1 Introduction

Cannabis (*Cannabis sativa* L.) is an annual herbaceous plant that has been used for various purposes (e.g., fiber, oil, medical, nutrition, ritual or ceremonial, and recreation) for thousands of years (1–3). Cannabis contains over 480 identified substances including more than 100 phytocannabinoids (1, 2), 120 terpenoids, flavonoids, omega-3- and omega-6 fatty acids, alkaloids, and other substances (1, 2, 4–21). Among the phytocannabinoids are the psychoactive (-)-*trans*-Δ^9^-tetrahydrocannabinol (Δ^9^-THC) and the non-psychoactive cannabidiol (CBD) (6, 7). Despite the abundance of potential pharmacologically active substances in *C. sativa* L., only two cannabinoids have been approved by the US Food and Drug Administration (FDA) for therapeutic use in humans. These FDA-approved products are Epidiolex^®^ (cannabidiol, Greenwich Biosciences, Inc., Carlsbad, CA) and Marinol^®^ (dronabinol, Solvay Pharmaceuticals, Marietta, GA). Furthermore, recent legislation has increased the use of cannabinoids in both humans and animals due to changes in the regulation of hemp.

The Agricultural Improvement Act of 2018 (commonly known as the 2018 Farm Bill) defined hemp as *C. sativa* L. containing < 0.3 percent Δ^9^-THC on a dry weight basis and removed hemp and hemp-derived products from Drug Enforcement Administration Schedule I status under the Controlled Substances Act. Thus, the 2018 Farm Bill marked a profound change in the regulation of cannabis in the US, established a pathway for the cultivation and interstate distribution of hemp-derived products that led to a substantial increase in the commercial availability of hemp products formulated for administration to pet animals. Consequently, results of the findings from pharmacokinetic studies investigating the disposition of CBD and its metabolites in dogs and cats have been reported (22–41). However, only one study has included a description of the pharmacokinetics of CBD in the same dogs after both IV and PO administration (42). Therefore, total clearance, volumes of distribution, elimination half-life after IV administration, and the extent of systemic availability after PO administration have not been subsequently determined although more sensitive methods of analysis for CBD and its metabolites have been implemented.

The endocannabinoid system (ECS) is a homeostatic neuroregulatory network of endogenous signaling lipids (endocannabinoids), cannabinoid (CB) and non-cannabinoid G-protein (GP) coupled receptors (e.g., CB1R, CB2R, GPR 55, and GPR119), and enzymes that regulate endocannabinoid biosynthesis and catabolism (43–49). Cultivars of *C. sativa* L. produce hundreds of biologically active phytocannabinoids, terpenes, and flavonoids (24, 50). Commercially prepared cannabis extracts contain bioactive phytocannabinoids (e.g., Δ^9^ THC, CBD) marketed as “full-spectrum,” “broad spectrum,” or individual isolates that are promoted for the treatment of chronic osteoarthritis, epilepsy, neuropathic pain, emesis, behavioral abnormalities, and skin diseases (51, 52). Full-spectrum products typically contain the psychoactive cannabinoid Δ^9^-THC, < 0.3 percent by US law, and non-psychoactive cannabinoids (e.g., CBD, cannabigerol (CBG), cannabichromene (CBC), other phytocannabinoids, and terpenes (51, 53). Broad-spectrum products contain non-psychoactive phytocannabinoids without Δ^9^-THC whereas isolates contain a single, highly purified phytocannabinoid (e.g., CBD). Differences in product formulation (i.e., phytocannabinoid constituents and their concentrations) are confounded by the interactions of phytocannabinoids with other *C. sativa* L. constituents including terpenes and flavonoids and by their effects on hepatic metabolism (i.e., CYP induction or inhibition) (54). For example, CBD modulates the effects of Δ^9^-THC through negative allosteric binding at CB1Rs and inhibition of various CYPs (e.g., CYP1A, CYP2B, CYP2D, and CYP3A) mediated metabolism provoking phytocannabinoid-drug interactions (25, 55–57). The variability in biological effects is due to product type, formulation, dose, route of administration, diet (e.g., food effect, fat source and its concentration), experimental conditions, and the recipient's health status. These factors create a highly complex scenario that necessitates properly designed, product dependent, and species specific pharmacokinetic and pharmacodynamic studies to identify and provide meaningful clinical guidance in their use (24, 26, 39, 41, 52, 58, 59). The primary objectives of this study were to investigate the pharmacokinetics and bioavailability of a full-spectrum Δ^9^-THC:CBD (1:40, w/w) product after intravenous (IV) and PO administration once and orally twice daily for 21 days. The secondary objective was to monitor selected oxidative metabolites and monitor physical, behavioral, hematologic, and blood chemical variables.

## 2 Materials and methods

### 2.1 Animal care and use

All animal procedures were approved by the East Tennessee Clinical Research institutional IACUC committee (PT-21-01). Animals were housed in the AAALAC-accredited facilities at the East Tennessee Research Center and maintained according to the Animal Welfare Act (60) and the *Guide for the Care and Use of Laboratory Animals* (61).

### 2.2 Dogs and outcome assessment

**Figure 1:**
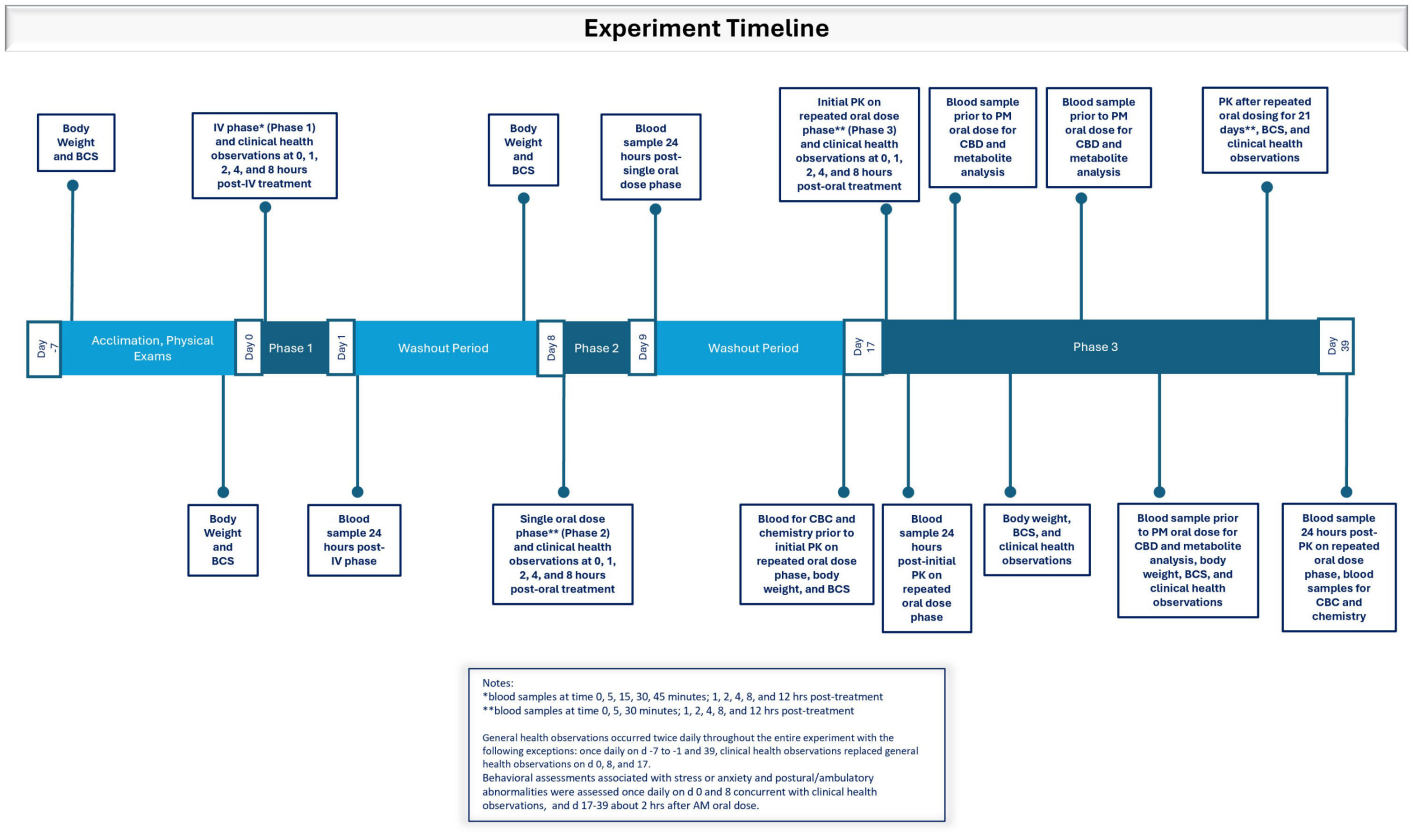
Timeline depicting experimental events during the acclimation period and Phases 1, 2, and 3 in which dogs were dosed with the test article dissolved in ethanol intravenously or orally in a small amount of wet dog food as a single dose or twice daily in an oral dosing regimen.

Six, purpose-bred, intact, 1–2-year-old beagle dogs (4 males, 2 females) weighing (mean; range) (10.7 kg; 7.5–11.2 kg) were used All dogs were individually housed and underwent a 7-day acclimation period before the study began. Diet, water, environment, and management were maintained throughout the study. A physical examination performed once during the acclimation period included body temperature, thoracic auscultation (heart and respiratory rate), and assessment of major organ systems (see Figure 1). Body weights were recorded on study days −7, −1, 7, 16, 24, and 31 (Figure 1). Body weights measured before each phase were used for dose calculations of CBD. A body condition score (BCS) was assigned on the mornings of study days −7, −1, 7, 16, 24, 31, and 38 using the Purina Body Condition Scoring system (https://www.purinainstitute.com; Figure 1).

Blood samples were obtained for a hemogram and blood chemical analysis on days 16 and 39 (Figure 1). Hematologic analysis included total leukocyte count, differential leukocyte count (percentage and absolute), packed cell volume, erythrocyte count, mean corpuscular volume, mean corpuscular hemoglobin, mean corpuscular hemoglobin concentration, hemoglobin concentration, and thrombocyte count. Blood chemical analysis included total protein, albumin, albumin/globulin ratio, globulin, blood urea nitrogen (BUN), BUN/creatinine ratio, creatinine, gamma glutamyl transferase, phosphorus, total bilirubin, alkaline phosphatase, alanine aminotransferase, aspartate aminotransferase, cholesterol, creatine phosphokinase, glucose, lactate dehydrogenase, sodium, potassium, sodium/potassium ratio, calcium, and chloride.

All dogs were fed Purina Pro Plan Adult Complete Essentials Shredded Blend Chicken & Rice dry dog food (minimum 18.2% crude fat, 29.5% crude protein dry matter basis) twice daily during the acclimation period and throughout the study. Dogs were fed immediately after each PO dose in the single and multiple dose phases. Water was available *ad libitum*.

Approximately 10–20 g of canned dog food^1^ (minimum 31.8% crude fat, 36.4% crude protein dry matter basis) was fed during Phases 2 and 3 to deliver the test article.

General health observations were recorded twice daily throughout the study with the following exceptions: once daily on study days −7 to −1 and study day 39, and clinical health observations replaced general health observations on study days 0, 8, and 17 (Figure 1). During general health observations, dogs were observed for signs of abnormal health and fecal consistency was scored (7-point scale). During clinical health observations, dogs were observed for signs of salivation, lethargy, tremors, diarrhea or vomiting, and unusual behavior. On days when clinical health observations were recorded, dogs were observed before treatment administration and at 1-, 2-, 4-, and 8-h post-treatment (± 5 min through 4 h; ± 15 min for 8 h post dosing). Abnormal health observations after administration of the test article were considered an adverse event.

Behavioral assessments associated with stress or anxiety and postural/ambulatory abnormalities were assessed once daily on study days 0 and 8 concurrent with clinical health observations, and on study days 17–39 (Phase 3) approximately 2 h after the morning PO dose (Figure 1). Behavioral assessments included hyperactivity, salivation, cowering/fearful posture, pacing, aggression, vocalization, panting, vomiting/atypical elimination, hypervigilance, shaking, and self-trauma or mutilation (e.g., tail biting, nail biting, flank sucking). The assessment scale was 0–5, with 0 = not present and 5 = extensive amount/severe. Postural and ambulatory assessments were assessed on a scale of 0–6, with 0 = no effect and 6 = lies prostrate on the floor.

### 2.3 Test article

The test article was prepared in a single batch from a fully characterized “full-spectrum” extract of *C. sativa* L. at a nominal CBD concentration of 100 mg/mL in medium-chain triglycerides (MCT) oil from coconut oil by a 3^rd^ party-contractor. The test article used in the study was analyzed by an ISO/IEC 17025 accredited commercial laboratory.^2^ The test article contained the cannabinoids CBD (100 mg/mL), CBC (3.08 mg/mL), Δ^9^-THC (1.53 mg/mL), CBDV (1.41 mg/mL), CBG (0.334 mg/mL) and CBN (0.107 mg/mL), and the terpenes (-)-alpha-bisabolol (10.6 mg/mL), beta-caryophyllene (5.24 mg/mL), (-)-guaiol (3.18 mg/mL), (-)-caryophyllene oxide (3.02 mg/mL), humulene (2.05 mg/mL), nerolidol (0.67 mg/mL), and limonene (0.48 mg/mL). No acidic cannabinoids were detected above the limit of detection (~0.001%). No heavy metal or solvent residues, mycotoxins, or pesticides were detected in the test article.

The IV dose was prepared for each dog by dissolving the requisite volume of the test article in 70% ethanol and filtering through a 0.22 μm syringe nylon filter immediately before IV administration. The IV dose of CBD (measured in μL of the test article in ethanol) was administered by bolus injection into a non-catheterized cephalic vein.

To prepare the oral CBD dose for each dog at each dose administration, the required volume of the test article (approximately 220 μL per dog per dose) was measured using a high-precision 1,000 μL micropipette. This volume was then dispensed into 10–20 g of canned dog food and thoroughly mixed to ensure even distribution. The time of administration (i.e., the time at which the dog had consumed the entire dose), the dose of CBD, the volume of the test article, and the weight of the canned food were recorded for each dose. The dry food (Purina Pro Plan Adult Complete Essentials Shredded Blend Chicken & Rice) was offered to each dog after it had consumed the medicated canned food.

### 2.4 Study design

The study design was a non-randomized, unmasked, three-phase study to determine the IV and PO pharmacokinetics of CBD in MCT oil in six beagle dogs. The dose rate of CBD was 2.2 mg/kg at each dose. The same dogs were used in all three phases of the study. Target analytes included CBD, Δ^9^-THC, 7-OH-CBD, 7-COOH-CBD, 11-OH-THC, and 11-COOH-THC. Dogs were observed twice daily during each treatment phase of the study and were fed to maintain a healthy weight and body condition score throughout the study.

Phase 1: Each dog was administered the test article IV diluted in ethanol. The dose rate of CBD was 2.2 mg/kg. Blood samples were collected from the opposite catheterized cephalic vein at 0, 5, 15, 30, 45 min and 1, 2, 4, 8, and 12 h post-treatment for determination of serum analyte concentrations (Figure 1). Phase 1 was followed by a 7-day washout period.

Phase 2: Each dog was administered the test article PO once in canned dog food as previously described. Blood samples were collected at 0, 5, 30 min and 1, 2, 4, 8, and 12 h after dosing. Phase 2 was followed by a 7-day washout period.

Phase 3: Each dog was administered the test article PO twice daily (every 12 h) for 21 consecutive days in canned dog food as previously described. Blood samples were collected on the first (study day 17) and last (study day 38) days of treatment on the same collection schedule as for Phase 2 (Figure 1). For the evening PO dose on study day 17 and study day 38, the 12-h blood collection occurred immediately before administration of the dose. Blood samples were collected immediately before the PM PO dose on study days 21, 26, and 31 (days 5, 10, and 15 after initiation of Phase 3) for determinations of all target analytes (Figure 1). All dogs were returned to the facility colony after the study (i.e., study day 39) and were managed in compliance with testing facility procedures.

### 2.5 Blood sample collection and preparation

Intravenous catheters were inserted into a cephalic vein after sedation with IM butorphanol (0.2 mg/kg) before blood collections on study days 0, 8, 17, and 38. The catheters were secured by bandaging, and patency was maintained with heparinized saline flush solution. Elizabethan collars were applied, and the subjects were observed frequently to prevent damage to the catheters. Catheters were removed after collection of the T+12-h sample.

Approximately 3 mL of blood was aspirated from the catheter using a 3-mL syringe, or by direct venipuncture using a 20 ga 1-inch needle (62). The time of each sample collection was recorded on the PK Sample Collection Record.

The blood was dispensed into an evacuated serum tube (red top) containing no anti-coagulants immediately following collection. Each tube remained at room temperature for ~1 h or in a water bath at ~38°C for a similar interval to facilitate clotting. The tubes were centrifuged at approximately 3,600 g for 10 min at room temperature. The serum from each tube was aspirated with disposable pipettes, divided equally between two cryovials, and frozen at −80°C. Collection tubes and cryovials were labeled with the study number, dog I.D., day or date, nominal sampling interval, and sequence (i.e., 1 of 2, 2 of 2). Collection data were recorded on the Serum Processing Record.

### 2.6 Sample analysis

Serum sample analyses for CBD, Δ^9^-THC and two metabolites of each were performed by a liquid chromatography-mass spectrometry method developed and validated for pharmacokinetic studies in dogs. The serum concentrations of all analytes are reported as total concentration (i.e., free plus protein-bound concentrations). See the Supplementary material for details of the method.

### 2.7 Method validation study

The method was validated based on the *M10 Bioanalytical Method Validation and Study Sample Analysis Guidance for Industry*.^3^ The study determined the specificity and robustness, the limit of detection (LOD), the limit of quantitation (LOQ), linearity and the linear range, accuracy, repeatability and intermediate precision, and dilution integrity. The results of the method validation study are reported in the Supplementary material.

### 2.8 Pharmacokinetic analysis

Non-compartmental pharmacokinetic analysis was used to determine the pharmacokinetics of CBD whereas compartmental pharmacokinetic analysis was used to determine the pharmacokinetics of Δ^9^-THC after single IV and PO because conventional compartmental models could not be fit to the data. Total clearance (CL_T_), volumes of distribution (compartmental volumes and steady-state volume of distribution), macro- and micro-constants, and half-lives of CBD and Δ^9^-THC after IV administration and absorption rate, lag time, and extent of systemic availability after PO treatment were determined by compartmental methods. Non-compartmental pharmacokinetic analysis included determination of AUC_t_ using the log-linear trapezoidal method with linear interpolation using pharmacokinetic analysis software (PKSolver, Department of Pharmaceutics, China Pharmaceutical University, E-mail: pksolver@cpu.edu.cn). Non-compartmental pharmacokinetic parameters for CBD and metabolites included the maximum serum concentration (C_max_), time at which C_max_ was observed (T_max_), lambda z (λ_z_), AUC_0_-_t_, and terminal half-life (t_1/2_). C_max_ and T_max_ were determined from model calculations or non-compartmental model analysis.

Based on a previous report for estimating the hepatic extraction ratio ER for CBD in dogs (42), the hepatic extraction ratio ER for each dog was estimated from its CL_T_ (assuming that CL_T_ represents hepatic clearance since hepatic metabolism of CBD is extensive and renal clearance of CBD is negligible), its hematocrit (HCT), and an estimate of liver blood flow in dogs (i.e., 30–45 mL/min/kg) (63, 64). The hepatic serum flow rates were estimated by multiplying the hepatic blood flow by (1-HCT) where HCT was the average of two determinations for each dog. The CL_T_ determined for each dog was then divided by the hepatic serum flow to obtain the estimate of the hepatic ER for each dog.

Serum concentrations of 11-OH-THC, and 11-*nor*-9-COOH-THC were less than LOQ in all samples collected after Treatments 1 and 2. Serum concentrations of CBD, 7-OH-CBD, 7-COOH-CBD, and Δ^9^-THC were above the LOQ, and are reported. AUC values and half-lives of metabolites were calculated where sufficient data were available.

The accumulation ratio AR during the multiple dose PO trial was estimated using AUC_0 − 12*h*_ data from the first dose and the next to last dose in the multiple dosing regimen as follows (65, 66):


$$\begin{array}{l} AR=\frac{{AUC}_{0-12\;h,\;next\;to\;last\;dose}}{{AUC}_{0-12\;h,\;first\;dose}} \end{array}$$

### 2.9 Statistical analysis

Statistical analyses were performed using statistical analysis software^4^ to determine statistical significance (*p* ≤ 0.05).

Selected complete blood count and serum chemistry test results measured immediately before the first dose and immediately after the last dose in Phase 3 were analyzed using two-tailed paired *t-*tests. Clearances in female and male dogs were compared using the two-tailed unpaired *t-*test. AUC values of CBD for 12 h after the first and last dose and ALP concentrations before and after the multiple dosing regimen (Phase 3) were compared by the two-tailed paired *t*-test to determine significance. The normality of residuals was assessed by the Shapiro-Wilk and the Kolmogorov-Smirnov tests.

A Student's *t*-test for paired samples was performed to assess differences between pharmacokinetic parameter estimates after PO and IV administration, and the Kolmogorov–Smirnov test was used to determine normality of the data. A non-parametric Wilcoxon signed-rank test for paired samples was used for T_max_, which is not a continuous variable, and was not normally distributed.

Serum concentrations of analytes collected at different times during Phase 3 were compared using repeated measures one-way ANOVA with multiple comparisons using Tukey's test to correct for multiple comparisons. Mean differences were considered statistically significant at *P* ≤ 0.05.

## 3 Results

No differences in body weight or body condition scores were observed during the study. The mean body weight on day −2 was 10.7 kg and on day 34 was 10.6 kg. All dogs consumed their food, including canned food mixed with the test article in Phases 2 and 3.

### 3.1 Hemogram and serum chemistry

**Figure 2:**
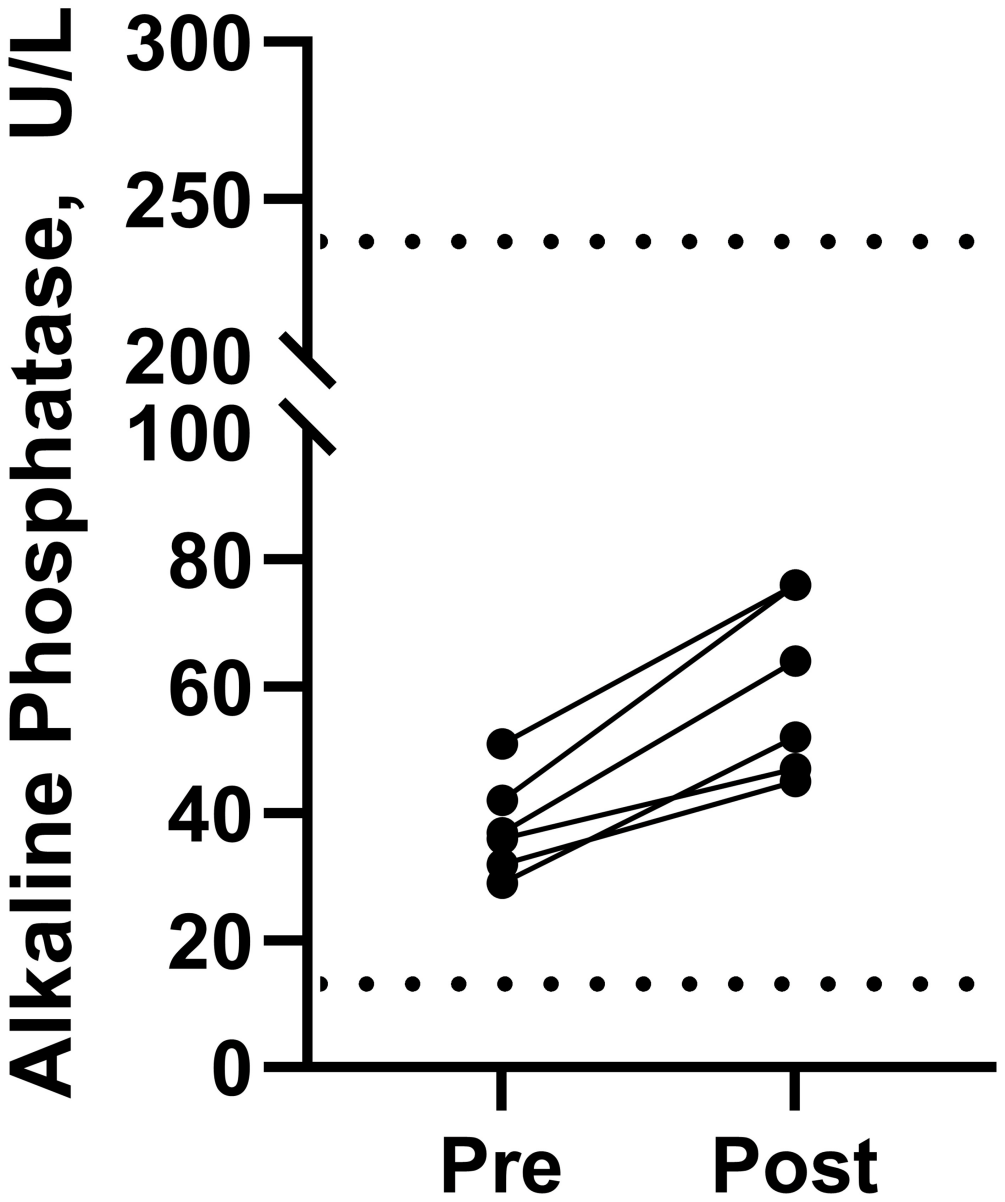
Serum alkaline phosphatase concentrations in beagle dogs immediately before (Pre) and immediately after the last dose (Post) in Phase 3 (2.2 mg of cannabidiol orally every 12 h). Normal limits for alkaline phosphatase in dogs are shown by the dashed lines (····) at 13 and 240 U/L.

The hemogram and serum biochemistry values were within normal ranges on day 16 (before starting Phase 3) and on day 39 (end of Phase 3). Alkaline phosphatase (ALP) was elevated (*P* = 0.0016) in all dogs at the end of Phase 3 compared to the day before the start of Phase 3 (Figure 2) although all values remained within the reference range. The ALP ranged from 29–51 U/L with a median value of 36.5 U/L on the day before the start of Phase 3 (i.e., day 16). At the end of Phase 3 (i.e., day 39) it ranged from 45–76 U/L, with a median value of 58 U/L. The mean and the 95% confidence interval for the increase in ALP were 22.2 U/L and 13.0–31.3 U/L, respectively.

### 3.2 Body condition, behavioral, and adverse events

The mean body condition score was 5.2 on study day −1 and 5.1 on study day 38. The test article produced no effects on postural and ambulatory measurements as all were assigned a score of 0. Some changes in behavior were noted during the study. Mild salivation was reported in three dogs in Phase 1, 1 and 2 h after IV injection and one dog 4 h after IV injection. Salivation was noted in one dog in Phase 2, 1 h after PO dosing, but no other behavioral changes were reported at 2, 4, or 8 h after PO dosing in Phase 2. A transient period of pacing was noted in one dog in Phase 3, 8 h after PO dosing but no other behavioral changes were reported at 1, 2, or 4 h after dosing.

Seven adverse events involving observation of mucus in feces, unformed feces, and regurgitation or vomiting were recorded during the study. On the day after the IV injection, blood and mucus were observed in the feces of one dog and mucus was observed in the feces of two dogs. On study day 9, 2 days after the single PO dose in Phase 2, unformed fecal matter was noted in one dog. On study day 17, on the first day of dosing in Phase 3, one dog regurgitated 1 h after dosing. During the repeated PO dose study, one dog vomited on the 9^th^ day and one dog produced unformed feces on the 12^th^ day.

### 3.3 Pharmacokinetic study

#### 3.3.1 Serum CBD, Δ^9^-THC, and metabolites after single dose intravenous administration of the test article–Phase 1

Serum concentrations of CBD, 7-OH-CBD, 7-COOH-CBD, Δ^9^- THC, 11-OH-THC, and 11-*nor*-COOH-THC after IV administration of CBD at a dose of 2.2 mg/kg BW were determined by a validated LC-MS/MS method. CBD and 7-COOH-CBD were detected in all samples collected after IV dosing, and 7-OH-CBD was detected in all samples except for 2 of 6 samples collected 24 h after IV dosing. Furthermore, Δ^9^-THC was detected in serum samples from all dogs and most samples except samples collected at 5 min and 12 and 24 h after IV dosing (which contained Δ^9^-THC). 11-OH-THC was quantified (LOQ 0.2 ng/mL) in all samples collected through 8–24 h after IV dosing, and 11-*nor*-9-COOH-THC was not detected (LOQ 1 ng/mL) in any sample collected after IV dosing of the test article.

**Figure 3:**
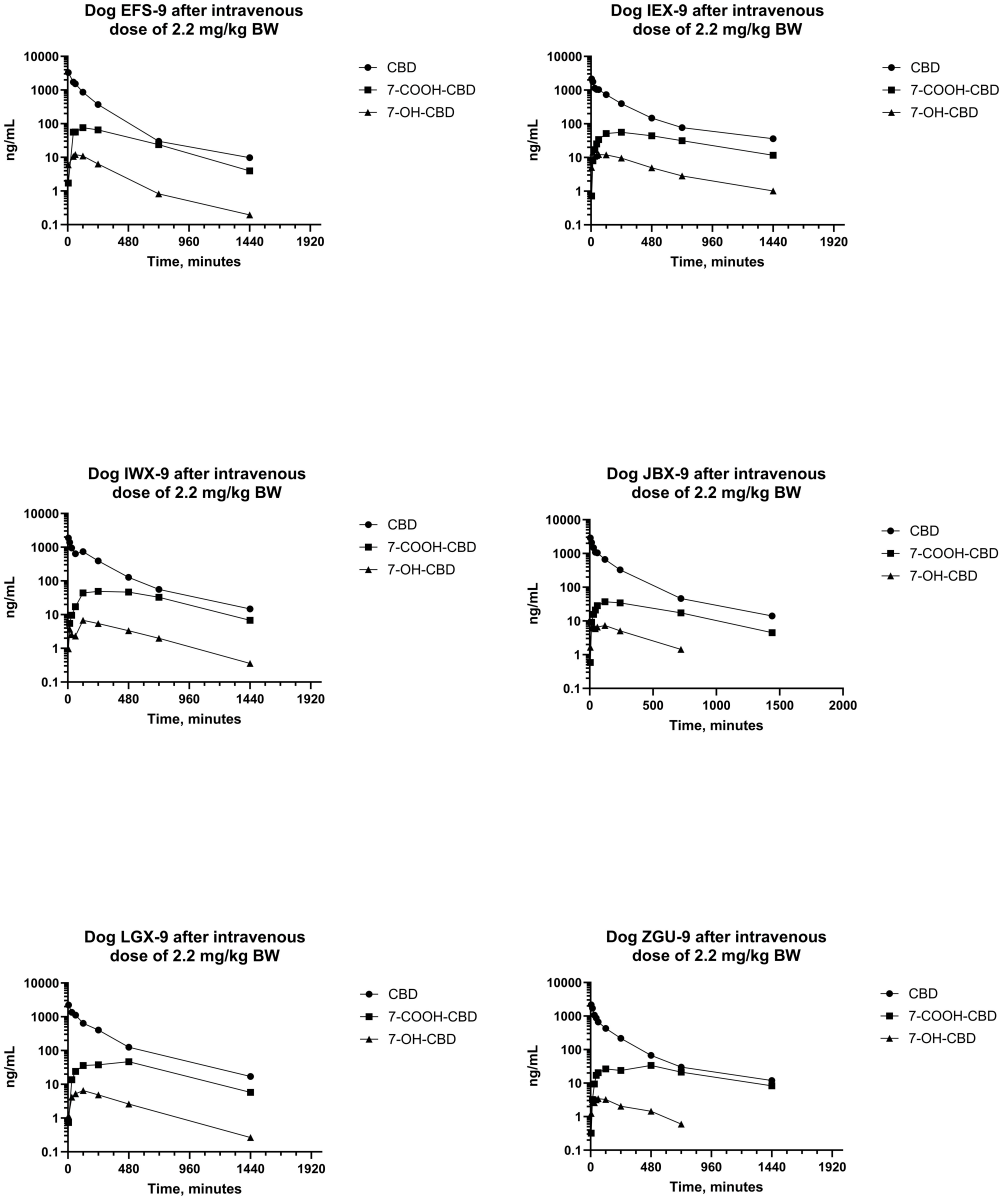
Plots of log serum concentrations of CBD, 7-OH-CBD, and 7-COOH-CBD after a single intravenous dose of CBD to six beagle dogs at a dose of 2.2 mg of CBD per kg BW.

**Figure 4:**
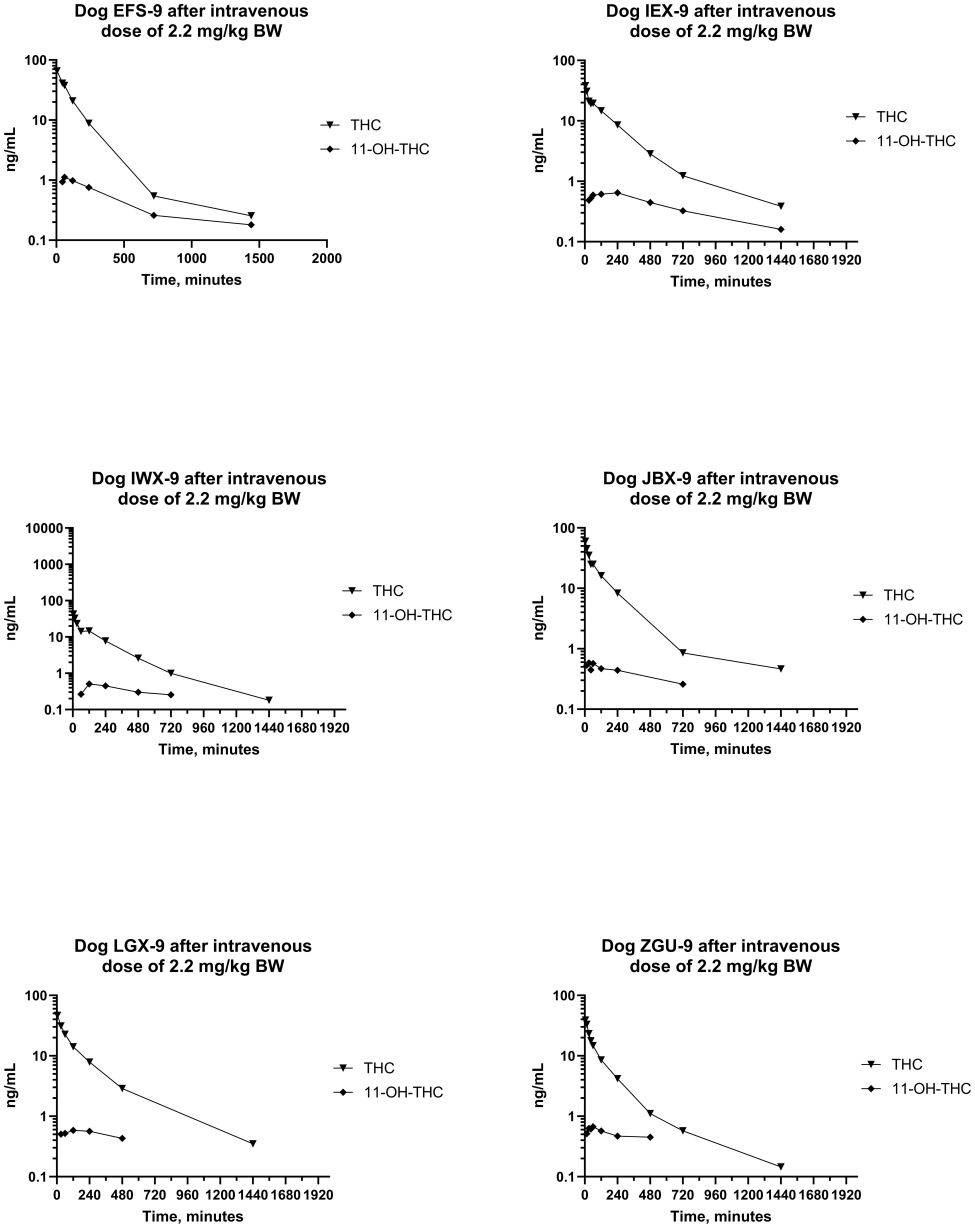
Plots of log serum concentration of Δ^9^-THC and 11-OH-THC after a single intravenous dose of the test article at a 2.2 mg of CBD per kg and 54 μg of Δ^9^-THC per kg BW.

Plots of log serum concentrations of CBD, 7-OH-CBD, and 7-COOH-CBD vs. time after single IV administration of CBD at a dose of 2.2 mg/kg BW are reported in Figure 3. Furthermore, plots of log serum concentrations of Δ^9^-THC and 11-OH-THC vs. time after single IV administration of 54 μg/kg BW Δ^9^-THC are reported in Figure 4.

Pharmacokinetic parameter estimates [median (range)] for CBD after IV administration are reported in Table 1. The disposition of CBD in beagle dogs after an IV dose of CBD at 2.2 mg/kg BW was characterized by a CL_T_ of 7.06 (6.14–10.5) mL/min/kg BW, V_ss_ of 2,130 (1,102–2,851) mL/kg BW, and terminal t_1/2_ of 291 (183–508) min. Maximum serum CBD concentrations ranged from 1,869–3,294 ng/mL, and CBD was still quantifiable 24 h after dosing. The MRT for CBD was 277 (179–429) min.

**Table 1:** Summary of non-compartmental pharmacokinetic parameter estimates after IV and PO administration of CBD at a dose of 2.2 mg/kg of body weight to beagle dogs (*n* = 6).

**Parameter**	**Units**	**Median**	**Minimum**	**Maximum**
**Single dose IV administration of CBD in test article diluted in**				
**ethanol**				
λ_z_	1/min	0.00241	0.00136	0.00379
t_1/2_	min	291	183	508
T_max_	min	5	5	5
C_max_	ng/mL	2.20 × 10^3^	1.87 × 10^3^	3.29 × 10^3^
C_0_	ng/mL	2.45 × 10^3^	2.17 × 10^3^	3.57 × 10^3^
AUC__0−∞_*obs*_	ng h/mL	5,196	3,486	5,972
MRT _0−∞_*obs*_	min	277	179	429
Vz__obs_	L/kg	3.11	1.62	6.26
Cl__obs_	mL/min/kg	7.06	6.14	10.5
V_ss, obs_	L/kg	2.13	1.10	2.85
ER	%	57	44	80
1-ER	%	43	20	56
**Single dose PO administration of CBD in test article mixed with**				
**moist dog food**				
λ_z_	1/min	0.00187	0.000888	0.00286
t_1/2_	min	420	243	781
T_max_	min	120	60	480
C_max_	ng/mL	270	118	537
T_lag_	min	0	0	4.88
AUC__0−∞*obs*_	ng h/mL	1,417	1,063	1,767
MRT__0−∞_*obs*_	min	549	387	655
V_z_/F__obs_	L/kg	14.7	8.28	30.4
Cl/F__obs_	mL/min/kg	26.3	20.8	34.5
F	%	31.2	17.8	35.7

Pharmacokinetic parameter estimates [median (range)] for Δ^9^-THC after IV administration of Δ^9^-THC in a “full-spectrum” formulation dosed at 54 μg/kg BW are reported in Table 2. The disposition of Δ^9^-THC in beagle dogs after an IV dose of 54 μg/kg BW was characterized by a CL_T_ of 8.85 (6.88–14.4) mL/min/kg BW, V_ss_ of 1,977 (1,304–2,305) mL/kg BW, and terminal t_1/2_ of 169 (139–476) min. The mean CL_T_ in females (8.32 mL/min/kg) was not different from that in males (7.05 mL/min/kg) (*P* = 0.424).

**Table 2:** Summary of two-compartment pharmacokinetic parameter estimates for Δ^9^-THC after IV administration and non-compartmental pharmacokinetic parameter estimates after PO administration to beagle dogs as a single dose of 54 μg/kg body weight.

**Parameter**	**Unit**	**Median**	**Minimum**	**Maximum**
**Single IV dose of** Δ^9^**-THC in test article diluted in ethanol**				
A	ng/mL	31.8	23.9	64.5
Alpha	1/min	0.0422	0.00963	0.0807
B	ng/mL	19.8	1.69	29.0
Beta	1/min	0.00409	0.00146	0.00498
k_10_	1/min	0.00902	0.00796	0.0119
k_12_	1/min	0.0170	0.00100	0.0349
k_21_	1/min	0.0188	0.00167	0.0419
½, alpha	min	17.4	8.59	72.0
½, beta	min	169	139	476
C_0_	ng/mL	51.4	44.6	70.3
V	mL/kg	1,052	768	1,209
CL	mL/min/kg	8.85	6.88	14.38
V_2_	mL/kg	867	488	1,197
CL_2_	mL/min/kg	15.7	0.8132	40.0
AUC_0−∞_	ng h/mL	102	62.5	131
AUMC	ng min^2^/mL	1.29 × 10^6^	0.602 × 10^6^	1.49 × 10^6^
MRT	min	203	160	230
V_ss_	L/kg	1.98	1.30	2.30
ER	%	56	43	91
(1-ER)	%	44	9.0	57
**Single PO dose of** Δ^9^**-THC in test article mixed with moist dog**				
**food**				
λ_z_	1/min	0.00153	0.000511	0.00423
t_1/2_	min	455	164	1,357
T_max_	min	120	60	480
C_max_	ng/mL	4.76	2.55	9.89
T_lag_	min	17.5	0	60
AUC _0−∞_*obs*_	ng h/mL	35.8	26.8	48.3
MRT _0−∞_*obs*_	min	610	263	1,473
Vz/F__obs_	L/kg	14.0	6.48	39.1
Cl/F__obs_	mL/min/kg	0.0253	0.0186	0.0335
F	%	40.9	20.5	46.2

Maximum serum Δ^9^-THC (i.e., C_0_) ranged from 44.6–70.3 ng/mL and Δ^9^-THC was still measurable 24 h after dosing. The MRT for Δ^9^-THC was 203 (160–230) min. Serum concentrations of 11-OH-THC were detected through 8–24 h after IV dosing of Δ^9^-THC in the “full-spectrum” formulation (see Figure 4).

#### 3.3.2 Serum CBD, Δ^9^-THC, and metabolites after single dose oral administration–Phase 2

**Figure 5:**
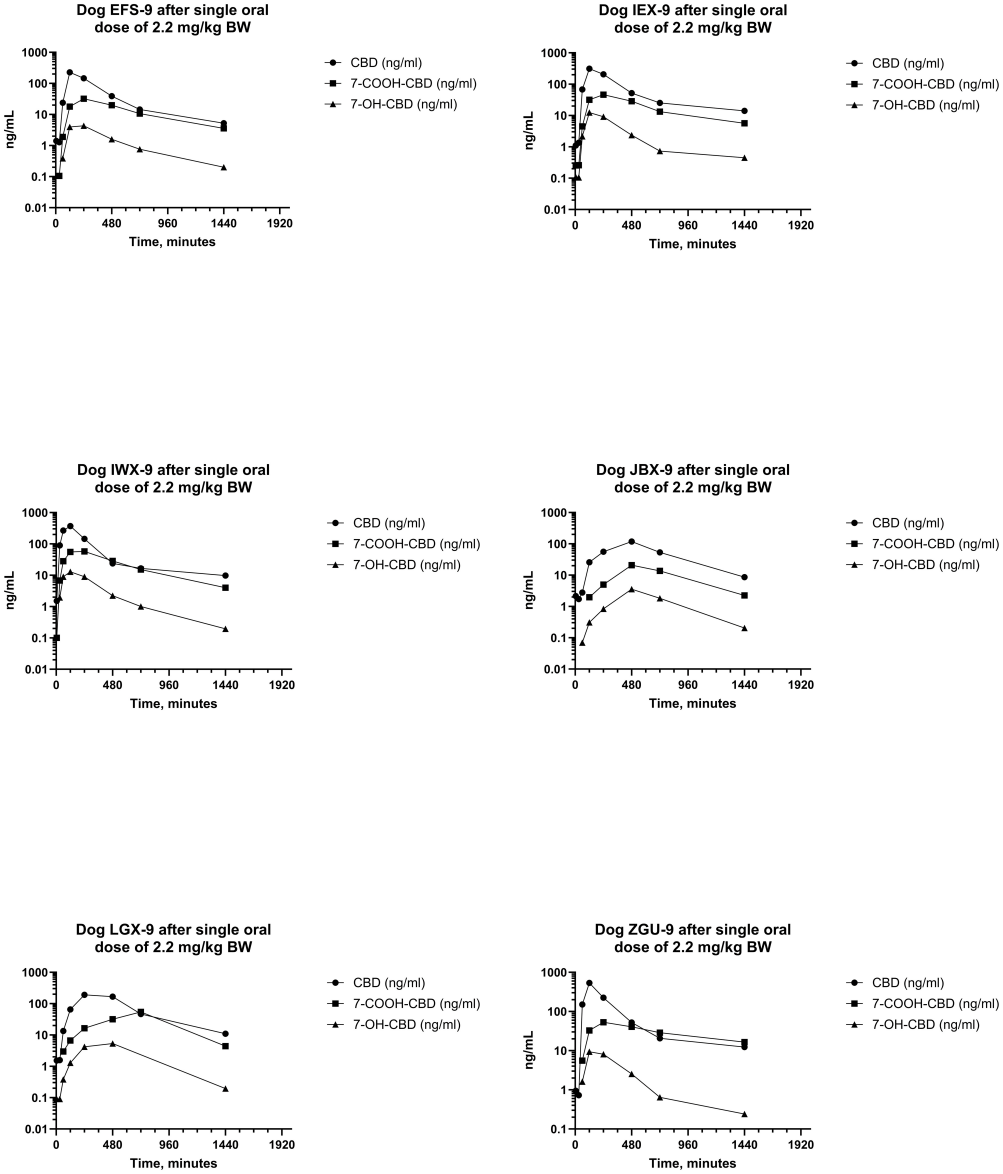
Plots of log serum concentrations of CBD, 7-OH-CBD, and 7-COOH-CBD after a single oral dose of CBD to six beagle dogs at a dose of 2.2 mg of CBD per kg BWt.

Serum concentrations of CBD, 7-OH-CBD, and 7-COOH-CBD after single PO administration of CBD at 2.2 mg/kg BW are reported in Figure 5 and pharmacokinetic parameter estimates are reported in Table 1. Maximum CBD ranged from 118–537 ng/mL (median 270 ng/mL) and were observed from 60–480 min (median 120 min) after dosing. The systemic availability of CBD ranged from 17.8%−35.7% (median 31.1%). CBD metabolites were quantifiable in all samples, 7-COOH-CBD concentrations were less than those of CBD but were greater than those of 7-OH-CBD at all sampling times, and the terminal concentrations of CBD and its metabolites decreased in parallel.

**Figure 6:**
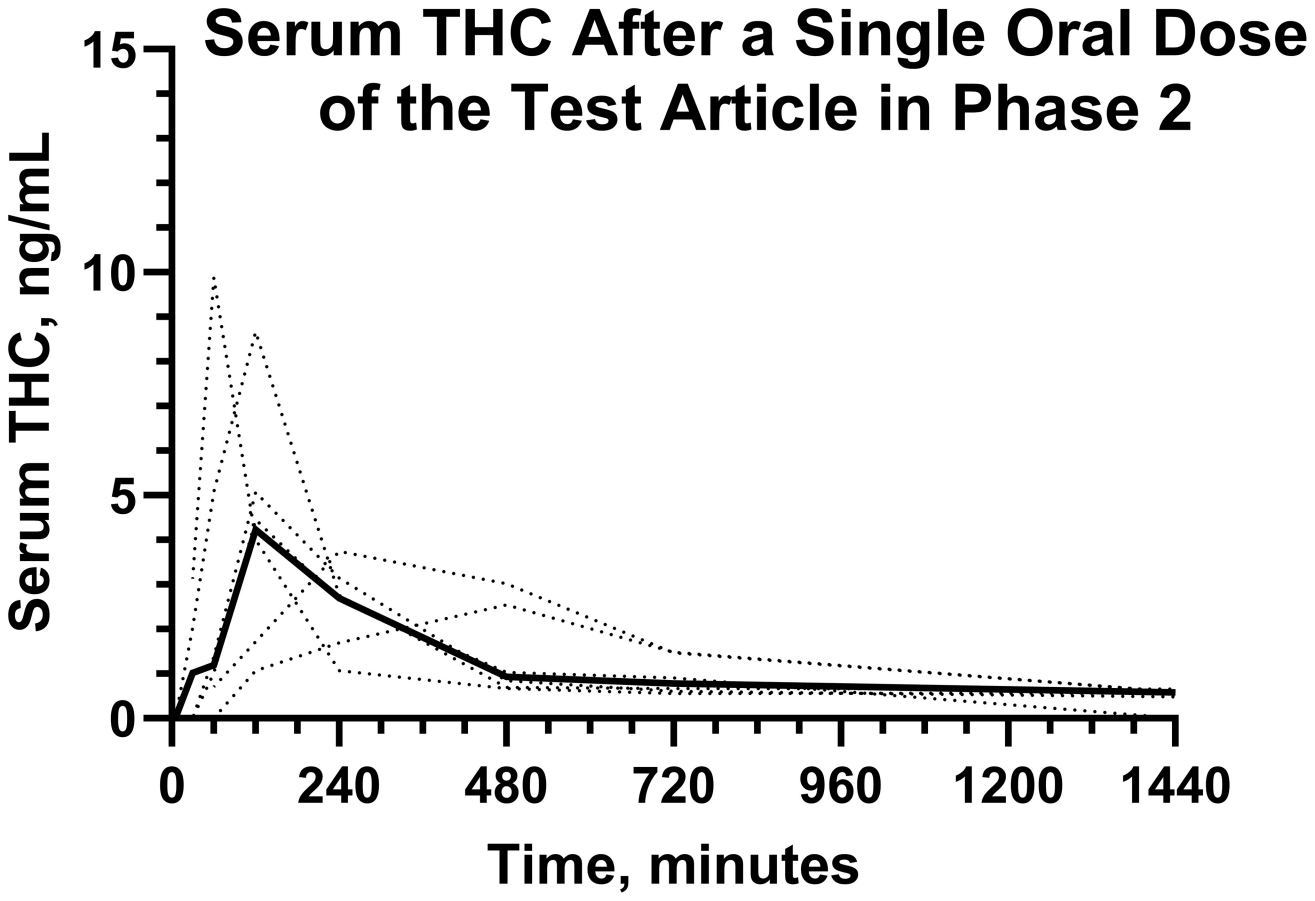
Individual (···) and average (—) serum Δ^9^-THC concentrations after a single dose of the test article in Phase 2.

Serum concentrations of Δ^9^-THC, after a single PO dose of Δ^9^-THC at a dose of 54 μg/kg BW are reported in Figure 6. Pharmacokinetic parameter estimates of CBD after IV and PO dosing are compared in Table 1. Selected pharmacokinetic parameter estimates for CBD, 7-OH-CBD, and 7-COOH CBD after IV and PO dosing are reported in Table 3. Serum concentrations of Δ^9^-THC were determined in all dogs after a PO dose of the test article, but 11-OH-THC and 11-COOH-THC were not quantifiable except that 11-OH-THC was detected in one dog from 2 to 24 h.

**Table 3:** Median (range) C_max_ and AUC_0 − t_ for CBD, 7-OH-CBD, and 7-COOH-CBD after single dose IV and PO CBD at 2.2 mg/kg body weight.

**Parameter**	**CBD**	**7-OH-CBD**	**7-COOH-CBD**
**CBD and metabolites after IV administration of CBD**			
C_max_, ng/mL	2,196 (1,870–3,290)	7.04 (3.42–14.40)	47.7 (33.7–76.2)
Relative C_max_, %	100	0.32 (0.18–0.44)	2.17 (1.80–2.32)
AUC_0 − t_, ng h/mL	5,105 (3,368–5,929)	54.8 (47.8–109)	639 (433–753)
Relative AUC, %	100	1.07 (1.42–1.84)	8.93 (6.06–9.04)
t½, min	291 (183–598)	278 (223–3,720)	404 (295–5,010)
**CBD and metabolites after PO administration of CBD**			
C_max_, ng/mL	270 (118-537)	7.35 (3.58–12.9)	49.7 (20.8–57.7)
Relative C_max_, %	100	2.91 (1.74–3.98)	15.2 (9.89–28.4)
AUC_0 − t_, ng h/mL	1,261 (1,032–1,689)	30.6 (24.5–66.8)	456 (204–536)
Relative AUC, %	100	2.43 (2.37–3.96)	36.2 (19.8- 31.7)
t½, min	420 (243–781)	269 (212–331)	

#### 3.3.3 Serum CBD, Δ^9^-THC, and metabolites after repeated dose oral administration–Phase 3

**Figure 7:**
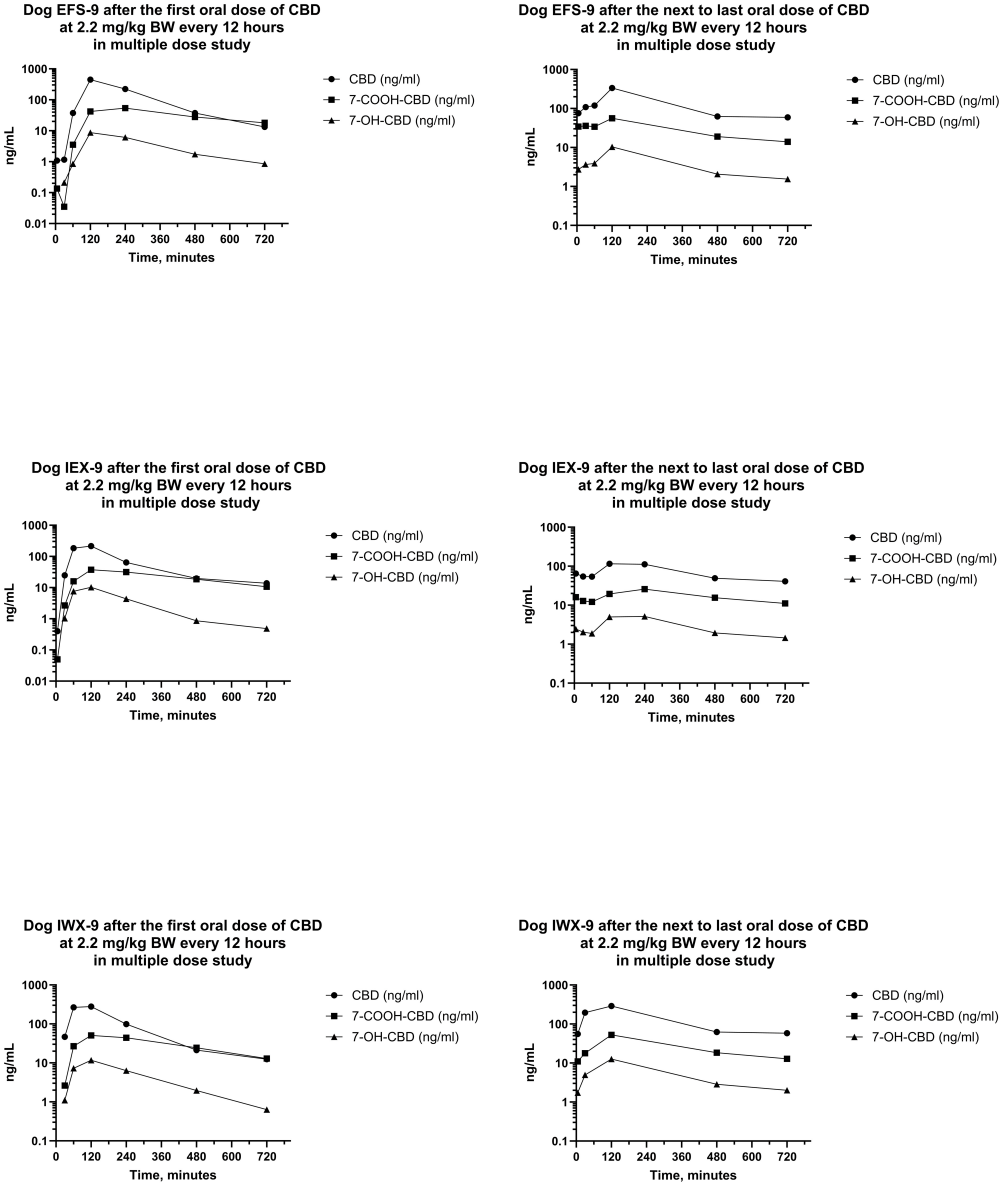
Plots of log serum concentrations of CBD, 7-OH-CBD, and 7-COOH-CBD after the first and last oral dose of CBD to three beagle dogs (EFS-9, IEX-9, and IWX-9) at 2.2 mg of CBD per kg BW every 12 h.

**Figure 8:**
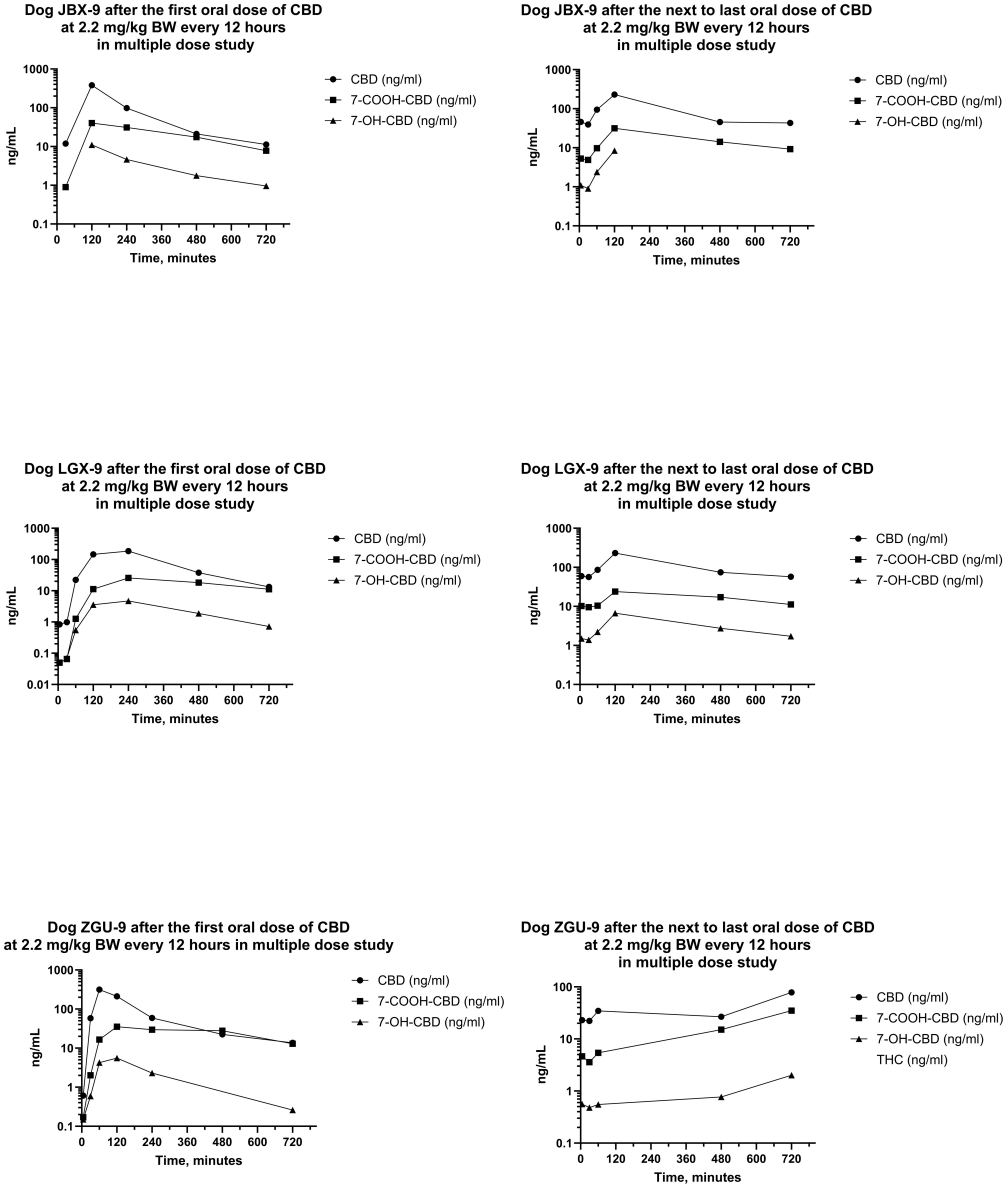
Plots of log serum concentrations of CBD, 7-OH-CBD, and 7-COOH-CBD after the first and last oral dose of CBD to three Beagle dogs (JBX-9, LGX-9, and ZGU-9) at 2.2 mg of CBD per kg BW every 12 h.

**Figure 9:**
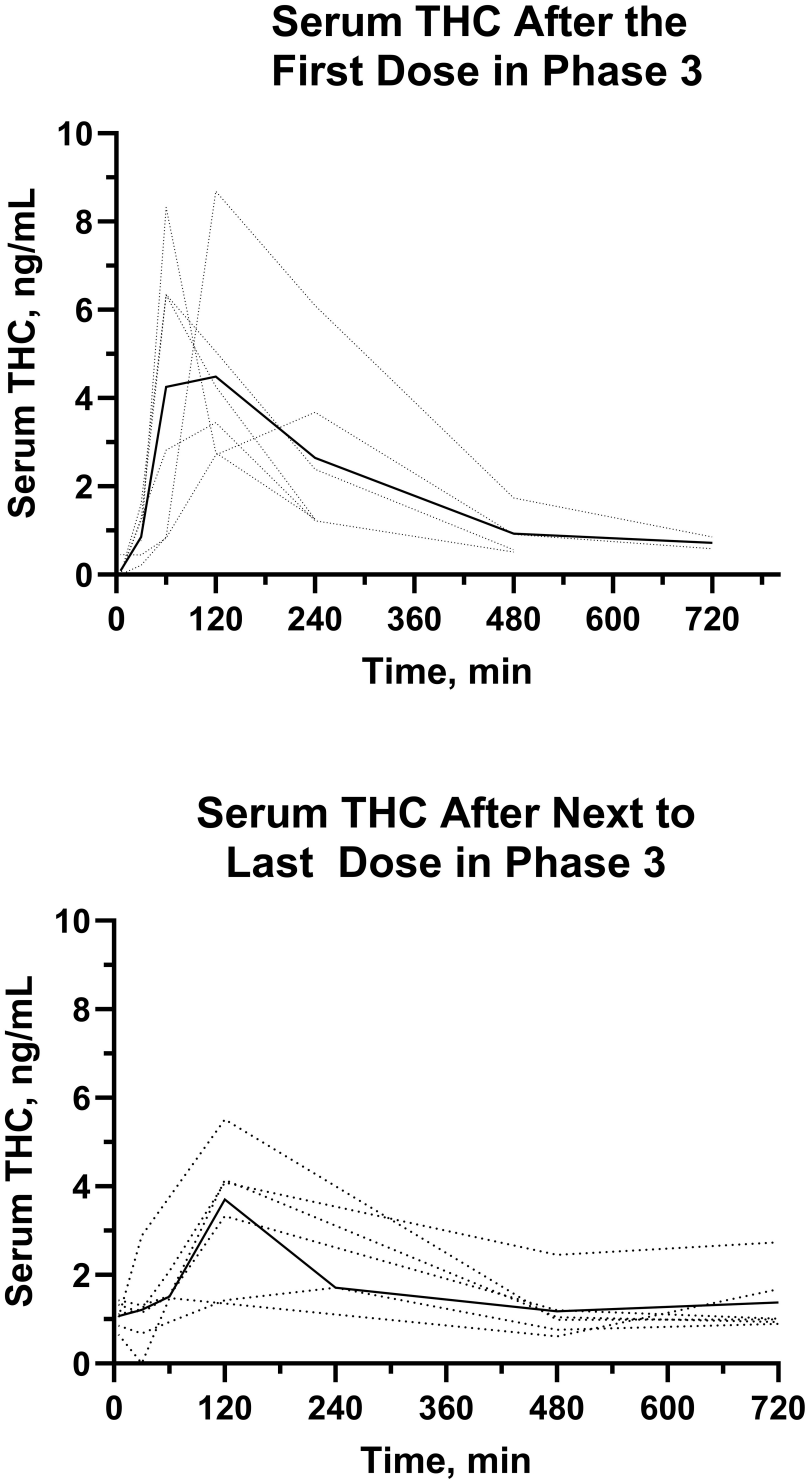
Individual (···) and average (—)Δ^9^-THC serum concentrations after the first and next to last dose in the multiple dose oral trial (Phase 3).

Serum concentrations of CBD, 7-OH-CBD, and 7-COOH-CBD after the first and next to last PO dose of CBD at 2.2 mg/kg BW every 12 h for 21 days are reported in Figures 7, 8. Serum concentrations of Δ^9^-THC after the first and next to last dose are reported in Figure 9.

Serum concentrations of CBD, 7-OH-CBD, 7-COOH-CBD, and Δ^9^-THC after the first dose were like those in the single dose PO study. Serum concentrations of 11-OH-THC and 11-*nor*-9-COOH-THC were quantifiable in some samples after the first dose, but all concentrations were < 1 ng/mL. Serum concentrations of CBD, 7-COOH-CBD, 7-OH-CBD, and Δ^9^-THC were present in all samples collected for 12 h following dose administration after the 20^th^ day, and 11-OH-THC was present at concentrations < 1 ng/mL in three of six samples.

Median (range) serum CBD concentrations in Phase 3 in samples collected 12 h after the first dose on day 1, and before dosing on days 5, 10, 15, and 21 were 13.3 (11.2–13.8), 143 (89.9–181), 127 (72.2–268), 109 (100–185), and 57.8 (41.1–78.3) ng/mL, respectively. Serum concentrations of CBD at 12 h after the first dose were lower than CBD concentrations 12 h after doses on days 5, 10, 15, and 21. Furthermore, serum CBD concentrations 12 h after the first dose of the day on day 21 were less than those collected 12 h after dosing on days 5 and 15. CBD concentrations on days 5, 10, and 15 after the beginning of the multiple dose study were not different.

The AUCs for CBD from the time of dosing through 12 h were determined after the first and the next to last dose during twice daily PO dosing of CBD for 21 days. The median (range) of the AUCs for 12 h after the first and next to last doses were 932 (723–1,408) ng min/mL and 1,193 (448–1,548) ng min/mL, respectively. The median (range) of the ARs was 0.880 (0.632–1.85). Furthermore, the median (range) of the serum CBD C_max_ was 298 (185–450) ng/mL and 230 (78–336) ng/mL, respectively, and did not differ between the first and next to last dose. On the other hand, the ratio of the t_1/2_ after the first dose to that after the last dose was 0.502 (0.436–0.540) indicating that it was about twice as long after twice daily dosing for 3 weeks than it was at the start of the multiple dosing regimen.

The AUC_0 − 12*h*_ for serum Δ^9^-THC from the time of dose administration through 12 hours were determined after the first and the next to last dose during twice daily PO dosing for 21 days. The median (range) AUC_0 − 12*h*_ were 21.0 (12.6–40.7) ng min/mL min and 22.4 (7.7–26.7) ng min/mL, after the first and next to last dose, respectively. The AUCs of Δ^9^-THC after the first and next to last dose were not different. The median (range) AR were 0.855 (0.574–1.26). The median (range) serum Δ^9^-THC C_max_ values after the first PO dose were higher (*P* = 0.01) than that after the next to last PO dose.

## 4 Discussion

To the authors' knowledge, this is the first study since the late 1980s including both IV and PO administration of CBD to the same dogs (42, 67). Serum CBD, 7-OH-CBD, 7-COOH-CBD, Δ^9^-THC, 11-OH-THC, and 11-COOH-THC were determined with a validated, tandem LC-MS^n^ method using stable isotope-labeled internal standards for each analyte. The method used in this study was more sensitive and included more target analytes than the HPLC-UV methods used in earlier studies (22, 27–29, 31, 34, 37, 41, 52, 68–72). Serum concentrations of Δ^9^-THC, 11-OH-THC, 11-COOH-THC, 7-OH-CBD, and 7-COOH-CBD were targeted in all phases of the study for a more complete characterization of the pharmacokinetics of orally administered CBD and Δ^9^-THC in dogs.

This study is the first to report CBD and two of its metabolites, as well as Δ^9^-THC and two of its metabolites, in serum samples from beagle dogs administered the test article via IV administration. The results of pharmacokinetic analysis after IV administration of the test article permitted estimation of the systemic availability of CBD after PO administration. These results indicated that the extent of systemic availability was consistent with estimates of ER obtained from the analysis of the corresponding IV data. The AUC data for 7-OH-CBD after IV and PO administration of CBD did not differ, thereby providing evidence that absorption of CBD from the gastrointestinal tract after PO dosing was complete (73). The t_1/2_ values of CBD, 7-OH-CBD, and 7-COOH-CBD were similar, indicating that the t_1/2_ values of metabolites are formation rate-limited.

Results of the pharmacokinetic investigations of CBD and Δ^9^-THC after IV and single PO doses are reported (Tables 1–3). Results of the pharmacokinetic analysis of IV CBD indicated CL_T_ near estimates of liver blood flow after estimating serum hepatic flow from the HCT for each dog. Estimates of the V_ss_ are consistent with extensive distribution of CBD and other cannabinoids. The median (range) of t½ after IV dosing was 291 (183–508) min, reflecting the effect of the large volume of distribution despite the rapid clearance. The disposition of Δ^9^-THC after IV administration was characterized by similar pharmacokinetic parameters.

Pharmacokinetic analysis of data from IV administration of the test article permitted estimation of the systemic availability of CBD and Δ^9^-THC after PO administration. These results indicated that estimates of the extent of systemic availability were consistent with estimates of ER and 1-ER obtained from the analysis of the corresponding IV data by equating CL_T_ of each substance to hepatic serum flow since CBD does not partition into erythrocytes (42). The systemic availability for CBD based on the estimate of 1-ER was 43% compared to the measured oral bioavailability of 31%. The systemic availability of Δ^9^-THC was estimated to be 44% based on an estimate of 1-ER under the same assumptions (64) and the measured availability was 41%. Although estimates of E (and 1-ER) were based on measurements of CL_T_ and HCT for each dog, the calculations require determination of hepatic blood flow which was not available for study dogs and therefore, estimated (30–45 mL/min/kg) (63, 64). Use of the published estimate of hepatic blood flow instead of actual hepatic blood flow in each dog contributed to the uncertainty in these calculations. Nevertheless, the estimates are in reasonable agreement with measurements based on comparisons of the AUC values after PO and IV dosing. In addition to the pharmacokinetic parameter estimates for CBD, the current study reports that AUC data for 7-OH-CBD after IV and PO administration of CBD did not differ, thereby providing evidence that absorption of CBD from the gastrointestinal tract after PO dosing was complete and was not limited by the poor aqueous solubility of CBD (73).

Plasma CBD concentrations in earlier studies were determined by HPLC-UV methods, in which the reported LOD was 25 ng/mL (42, 67, 69, 74–76). The pharmacokinetics of CBD in one frequently cited study were determined after single IV doses of about 45 and 90 mg to dogs weighing between 16 and 24 kg (~2.25 and 4.5 mg/kg, respectively) and a single PO dose of 180 mg (~9 mg/kg) to each of six mixed-breed dogs. The disposition of CBD was well characterized after IV administration, but serum metabolites of CBD were not targeted, and CBD was not detected in three of the six dogs after PO administration because the plasma concentrations were too low to be quantified. The results of that study indicated multiexponential disposition of CBD after IV administration, rapid distribution of CBD from plasma to tissues, rapid clearance approximately equal to hepatic plasma flow, large volumes of distribution, high ER of 0.74, dose-dependent AUC, and extensive first-pass metabolism after PO administration (42). The CL_T_ was rapid at 17.3 L/h and 15.9 L/h, respectively (approximately 14.4 mL/min/kg and 13.3 mL/min/kg, respectively (42). Furthermore, the V_ss_ values were 5.65 and 5.85 L/kg, respectively, the volumes of distribution beta (Vd_β_) were 8.4 and 10.5 L/kg, respectively, and the terminal t½ values were 6.8 h and 9.3 h, respectively (42).

Results from a companion study of the urinary excretion of CBD and its metabolites indicated that CBD is extensively metabolized, and that renal clearance of CBD is a minor route of elimination since only 0.013% of the administered dose was recovered from the urine. These findings lead to the conclusion that the CL_T_ is due to hepatic clearance (42, 67). Furthermore, the total amount of CBD and CBD-related substances recovered in the urine accounted for < 2% of the administered dose (67) indicating that metabolites are eliminated by other routes like biliary excretion or that a portion of the dose was eliminated in the feces. The metabolites identified in urine collected after oral dosing of CBD result from oxidation to mono-, di-, and tri-hydroxy and acid metabolites (67, 75, 76). Several of the alkyl side chain oxidized metabolites are subsequently conjugated with glucose and excreted in the urine (67, 75, 76).

The conclusions of other studies of the disposition of PO CBD in dogs attribute low systemic availability to poor absorption or extensive first-pass metabolism without any direct evidence. These studies did not acquire IV data and therefore could not estimate the extent of systemic availability from AUC after PO and IV administration. The results of the current study indicate modest first-pass metabolism of PO CBD in dogs, but do not show any evidence for poor or incomplete absorption. Peak serum CBD concentrations after PO administration were observed at 120 min (range 60–480) indicating relatively rapid absorption. Comparison of AUC values for CBD after PO and IV administration indicated that median (range) of the systemic availability was 40.9 (20.5–46.2)%, and a comparison (Table 3) of the AUCs for 7-OH-CBD after IV and PO administration of CBD indicated complete absorption of the dose in this study. Since the test article included CBD in MCT oil and was fed with canned dog food containing fats, the systemic availability of the CBD may have been improved relative to other studies because losses of undissolved CBD in the feces were unlikely. Results of studies of the disposition of CBD in dogs (and other species) indicate a substantial first-pass effect after PO administration and are consistent with predictions for highly lipophilic substances like CBD and other cannabinoids because lipophilicity and related factors determine, or have substantial influence on pharmacokinetic properties (77–79).

Natural cannabinoids are rapidly distributed into highly vascularized organs (e.g., lung, heart, brain, and liver) but are more slowly distributed into less vascularized tissues (e.g., adipose tissue) (80). The rapid distribution of CBD is reflected in the rapid reduction in CBD serum concentrations after IV administration in this study (Figure 3) and those in an earlier study (42). The extensive distribution of CBD into highly vascularized tissues and its subsequent partitioning into less vascular tissue results in a large fraction of the administered dose residing in tissues outside the blood. Furthermore, highly lipophilic tissues like adipose tissue have a large capacity for storing CBD from which it slowly partitions back into the blood as blood concentrations decrease. The partitioning of CBD into such tissues contributes to the large volumes of distribution that have been reported in this study (Table 1) and a previous study (42). The median (range) of V_ss_ in this study in beagle dogs was 2.13 (1.10–2.85) L/kg and the mean (SD) V_ss_ from the previous study was 5.65 ± 2.1 L/kg after a dose of 45 mg IV (42). Large volumes of distribution characterize the disposition of CBD in other species as well. In a study of older horses, the mean (SD) V_ss_ of CBD was 5.5 ± 1.5 L/kg, indicating extensive distribution in this species (81). Furthermore, in pigs, the mean (SD) V_ss_ was 3.3 (±0.29) L/kg (82). In humans, the volume of distribution of CBD after IV administration of its deuterium- labeled analog was reported as 2,520 L (approximately 36 L/kg, assuming a BW of 70 kg) (83). The extensive distribution of CBD and correspondingly large volumes of distribution are largely a consequence of the high lipophilicity of CBD.

Metabolism of CBD in dogs is extensive and only small amounts of CBD are excreted unchanged in the urine, so CL_T_ is attributed largely or exclusively to hepatic clearance (74–76). After the CL_T_ estimates based on plasma measurements in the previous IV study were converted to blood clearance, the plasma clearance estimates [i.e., 20.3 and 19.5 mL/min/kg, respectively (42)] were compared to the liver blood flow reported for dogs (63) to estimate the hepatic ER for CBD. The ER estimate of 0.74 (42) in that study predicted that approximately 26% of an oral dose of CBD to dogs escapes first-pass metabolism and reaches the systemic circulation unchanged (42). Due to the limitations of the analytical methodology (i.e., HPLC-UV with a reported lower limit of detection (LOD) for CBD of 25 ng/mL (69), CBD was not quantifiable after PO administration of 180 mg in three of the six dogs (42). The extent of systemic availability in the remaining three dogs was reported to be 13%, 13%, and 19% (42). These estimates are in reasonable agreement with the estimate of 26% from the prediction based on the ER and may have been lower due to assay limitations (42).

Pharmacokinetic parameter estimates after IV CBD at 2.2 mg/kg in the present study are reported (Table 1). Our findings and those from an earlier study using purified CBD in dogs are similar (42). In both cases, the disposition of CBD was characterized by non-compartmental modeling. The dose of 2.2 mg/kg was near the dose of 2.25 mg/kg in the earlier study, but the sensitivity and specificity of the CBD analysis in serum were improved by LC-MS^n^ analysis in the current study, thereby resulting in the measurement of serum CBD, Δ^9^-THC, and oxidative metabolites at lower concentrations than were achievable by the HPLC-UV method used previously. CBD concentrations decreased rapidly during the first 4 h after IV administration, then declined at a slower rate characterized by a median (range) t_1/2_ of 291 (183–508) min compared to 420 (294–888) min in the previous study (42). The rapid decline in CBD concentrations after dosing in the present study did not appear as pronounced as that reported in the previous study (42, 69) because sampling was less frequent during this period.

Comparisons of the plasma or serum clearance of CBD to the liver blood flow in the previous (42)and current study of IV CBD in dogs indicate that hepatic clearance is likely flow-rate dependent. Therefore, variability in liver blood flow rates will affect hepatic clearance more than variability in the free fraction and intrinsic metabolic activity (i.e., f_u_·CL_int_ where f_u_ is the fraction unbound and CL_int_ is the intrinsic clearance) (64, 84–88). The estimate of ER from the present study is important as it suggests that the hepatic clearance of CBD in dogs is likely flow rate-limited. Consequently, it is unlikely to be significantly impacted by changes in plasma protein binding and intrinsic hepatic metabolic clearance (88). Since the extent of systemic availability after PO administration of a high extraction ratio drug depends on 1-ER, small changes in ER can result in differences in systemic availability. This variability is likely responsible for the inconsistent systemic availability of orally administered CBD, as reported in this and other studies of the disposition of CBD in dogs (27–29, 31, 34, 37, 39, 41, 52, 72, 89, 90).

The estimate of hepatic extraction ratio provided an estimate of the extent of first-pass metabolism that agreed with the direct determinations based on AUC comparisons.

The metabolism of CBD has been investigated in mice (91–96), rats (74, 80, 94, 97), dogs (67, 74–76), cats (94), gerbils (94), guinea pigs (94), rabbits (94), hamsters (94), horses (81, 98–101), pigs (82), sheep, cattle (102), and humans (74, 103–106). Numerous oxidative and conjugated metabolites of CBD have been identified from urine after PO and IV administration to dogs (67, 71, 74–76), but this study is the first in which metabolites of CBD have been determined in serum after PO and IV doses. Serum concentrations of 7-OH-CBD and 7-COOH-CBD were determined by LC-MS^n^ and the resulting data were subjected to non-compartmental pharmacokinetic analysis. Serum concentrations and AUC of 7-OH-CBD were lower than those of 7-COOH-CBD and CBD in all samples after IV and PO administration of CBD (Figures 3, 5). Similarly, serum concentrations and AUC of 7-COOH-CBD were lower than those of CBD in all samples after IV and PO administration. The C_max_ values for serum 7-OH-CBD were observed somewhat earlier than those of 7-COOH-CBD suggesting that 7-OH-CBD was formed before 7-COOH-CBD and t_1/2_s of CBD, 7-OH-CBD, and 7-COOH-CBD were similar, indicating that the half-lives of metabolites are formation rate-limited (107–109). This conclusion is supported by the observation that the “ratio plot” (i.e., C_7 − OH − CBDt_/C_CBD_ and C_7 − COOH_/C_CBD_) vs. time after administration increased, as was previously described for formation rate-limited elimination (110).

In dogs and humans, CBD is sequentially metabolized to 7-OH-CBD, the major active metabolite, and then to 7-COOH-CBD, an inactive metabolite (106). The current finding for relative C_max_ and AUC values for CBD, 7-COOH-CBD, and 7-OH-CBD contrast with those from studies of PO CBD in humans in which serum 7-COOH-CBD concentrations are higher than those of both CBD and 7-OH-CBD (106). 7-COOH-CBD is a metabolite in horses where it attains higher plasma concentrations than CBD and 7-OH-CBD and has a longer t_1/2_ than CBD. Consequently, plasma 7-COOH-CBD is detectable after CBD has become undetectable in horses (81, 100, 101, 111, 112).

Pharmacokinetic parameter estimates for Δ^9^-THC after IV administration to beagle dogs from the present study were compared to those reported previously after normalizing volumes of distributions and clearances based on body weights of the dogs (64). Although a highly specific and sensitive LC-MS^n^ method was used to determine Δ^9^-THC in the present study, the use of ^14^C-labeled Δ^9^-THC, a larger dose, and more frequent and prolonged sampling in the previous study allowed detection of Δ^9^-THC and its metabolites for an extended period after dose administration. The model used to describe these data used five exponential terms, including one that described the disposition of Δ^9^-THC at lower concentrations and for a longer period than was possible in the present study. Consequently, the magnitudes of the half-lives and volumes of distributions were substantially different from the values reported in Table 2. For example, the mean V_c_ in the present study was 1,007 mL/kg whereas in the earlier study it was only 94.7 mL/kg. Furthermore, the median (interquartile range) of the β-phase t_1/2_ of Δ^9^-THC in the present study was 170 (141–253) min whereas it was 11,820 min (197 h) in the earlier study. On the other hand, the median (interquartile range) for CL_T_ in the present study was 8.52 (7.77–10.1) mL/min/kg and 8.85 (7.85–11.0) mL/min/kg from the earlier study. These values were not different.

Differences in pharmacologic responses of dogs and humans from exposure to Δ^9^-THC have been hypothesized to be due to differences in its metabolism (113) and differences in the metabolites excreted in urine (74). Serum concentrations of 11-OH-THC, a pharmacologically active metabolite of Δ^9^-THC were present after IV, but not after PO, administration in this study (Figure 4). This metabolite was present in the earliest samples collected, reflecting its rapid formation in dogs (64, 114). Serum concentrations of 11-OH-THC were always less than those of Δ^9^-THC and the ratio of serum concentration of Δ^9^-THC to that of 11-OH-THC decreased with time. Therefore, this finding suggests that this metabolite may contribute to the effects of CBD after IV exposure or dosing to dog.

The test article was well tolerated when administered as a single IV dose, a single PO dose, and repeated PO doses every 12 h for 21 days to beagle dogs (4 males, 2 females) weighing (median; range) 10.7 kg; 7.5–11.2 kg. Occasional salivation and mucus in the feces, unformed feces, and regurgitation or vomiting were observed in some dogs during different phases of the study. These results are consistent with other studies in which CBD isolates and full-spectrum CBD products have been administered in single or multiple PO doses to beagles and mixed-breed dogs (34, 58, 71).

An increase in ALP within the normal range was noted between the first and last doses after repeated PO administration of CBD at a daily dose of 4.4 mg/kg (2.2 mg/kg every 12 h) in this study (Figure 2). This finding is consistent with previous reports of elevated ALP after repeated PO administration of CBD to dogs at daily doses of 1–4 mg/kg (29), 1–12 mg/kg (31, 70), 4 mg/kg (71), 5 and 10 mg/kg (115), 5 mg/kg (116), 5–20 mg/kg (40), and 10–20 mg/kg (32). A previous investigation into the effects of CBD on hematologic and biochemical parameters in dogs after PO administration of CBD at 4 mg/kg daily for 6 months found that ALP was not elevated at the end of 2 weeks, but was elevated at the end of 4, 10, 18, and 26 weeks after which the ALP returned to pre-study values within the 4-week washout period (71). After daily dosing of CBD at 4 mg/kg, mean ALP concentrations were 98.0, 139, 145, 135, and 136 U/L after 2, 4, 10, 18, and 26 weeks, respectively (71). Furthermore, the ALP concentrations at 4, 10, 18, and 26 weeks were greater than the upper limit of the clinically normal range (i.e., 131 U/L) for the test method that was used (71). In addition to the elevated ALP concentrations, bone alkaline phosphate (BALP) concentrations were also elevated and strongly correlated with the ALP concentrations suggesting that the increased ALP was partially due to increased BALP (71). Although increased ALP is usually considered an indicator of dose-dependent drug-induced liver injury, the underlying pathophysiology associated with its rise during chronic treatment with CBD in dogs is unknown (117).

Limitations of this study include lack of a placebo group, small number of subjects, and the short duration of the repeated oral dose study period. The small number of subjects reduced the statistical power, and the short duration of the repeated dose study may have precluded determination of effects arising during longer treatment. Furthermore, since the study used purpose-bred, 1–2 year old, beagle dogs, the effects, if any, of breed, age, neutering, and sizes of the dogs were not investigated.

In conclusion, the disposition of CBD and Δ^9^-THC in beagle dogs is characterized by rapid and extensive hepatic metabolism resulting in a substantial first-pass effect after oral administration. Both CBD and Δ^9^-THC are highly lipophilic substances that are characterized by large V_ss_ (~2 L/kg), due to distribution into tissues such as fat from which they are slowly released. The test article was well tolerated when administered as a single IV dose, a single PO dose, and repeated PO doses every 12 h for 21 days to beagle dogs. Serum concentrations in the current study were determined by a sensitive and specific LC-MS^n^ method in contrast to earlier studies of the disposition of CBD in dogs after IV and PO administration (42, 67, 69). Serum concentrations of Δ^9^-THC, 11-OH-THC, 11-COOH-THC, 7-OH-CBD, and 7-COOH-CBD were determined in the current study for a more complete characterization of the pharmacokinetics of orally administered CBD in contrast to other studies (23, 26–28, 31, 33, 34, 37, 39–41, 52, 72, 118–120). Both CBD and Δ^9^-THC are subject to sequential first-pass metabolism to oxidative metabolites. Furthermore, we have investigated the pharmacokinetics of CBD and Δ^9^-THC after IV administration to the same dogs and characterized the pharmacokinetics of both. We postulate that the CL_T_ of both CBD and Δ^9^-THC in dogs is due to hepatic metabolism, that hepatic clearance is blood flow-rate dependent, and that the hepatic ER predicts the extent of systemic availability of both CBD and Δ^9^-THC after PO administration when fed with moist dog food.

## Data Availability

The raw data supporting the conclusions of this article will be made available by the authors, without undue reservation.

## Footnotes

^1^Purina One^®^ SmartBlend^®^ Chicken & Brown Rice Entrée.

^2^Kaycha Labs, 879 Federal Blvd., Denver, CO, USA.

^3^ https://www.fda.gov/media/162903/download

^4^GraphPad Prism version 8.3.1 for Windows, GraphPad Software, San Diego, California USA (https://www.graphpad.com).
