# Supplementary material for: Pharmacokinetics of cannabidiol, (-)-trans-Δ9-tetrahydrocannabinol, and their oxidative metabolites after intravenous and oral administration of a cannabidiol-dominant full-spectrum hemp product to beagle dogs
Source: Front Vet Sci. 2025 Apr 8;12:1556975. doi: 10.3389/fvets.2025.1556975 (PMC12013723; doi:10.3389/fvets.2025.1556975)
Supplement: Supplementary file 1 [file Data_Sheet_1.docx]

**Supplemental Material**

## Experimental Design

The experimental design was a non-randomized, unmasked study conducted in three phases to determine the intravenous (IV) and oral (PO) pharmacokinetics of CBD and THC in beagle dogs. In the first phase, the test article (“full-spectrum” CBD-dominant hemp extract in MCT oil) was dissolved in ethanol and administered IV to each dog at a dose of 2.2 mg of CBD per kg of body weight (BW). In the second phase, the test article was mixed with canned dog food and administered once PO to the same dogs at the dose rate of 2.2 mg of CBD per kg BW. In the third phase, the test article was mixed with canned dog food as before and administered PO every 12 hours at the dose rate of 2.2 mg of CBD per kg BW to the same dogs for twenty-one days. The timeline depicting all phases of the study is shown in Figure 1.

Figure 1. Timeline depicting experimental events during the acclimation period and Phases 1, 2, and 3 in which dogs were dosed with the test article dissolved in ethanol IV or PO in a small amount of canned dog food as a single dose or twice daily in the PO dosing regimen.


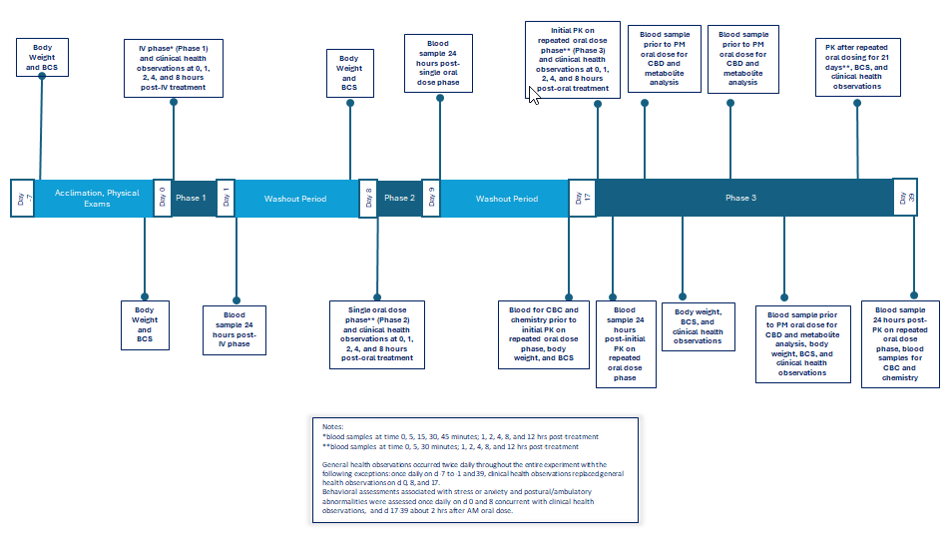
Method Validation and Performance

A validated liquid chromatographic-tandem mass spectrometric (LC-MS^2^) bioanalytical method was used to determine concentrations of CBD and Δ^9^-THC and their major metabolites 7-hydroxy-cannabidiol (7-OH-CBD), 7-carboxy-cannabidiol (7-COOH-CBD), 11-hydoxy-Δ^9^-tetrahydrocannabinol (11-OH- Δ^9^-THC), and 11-*nor*-9-carboxy-Δ^9^-tetrahydrocannabinol (11-COOH- Δ^9^-THC) in dog serum.

Stock solutions containing all analytes at 200 ng/mL or 1000 ng/mL in methanol were used to prepare calibrators (Supplement Table 1). The stock solutions were diluted in methanol to produce calibrators at concentrations of 0.200, 0.500, 1.00, 2.00, 5.00, 20.0, and 100 ng/mL of dog serum.

*Supplement Table 1. Dilution of standard solutions to prepare stock standard solutions to prepare calibrators.*

| Calibration solution | Analyte stock solution (ng/mL) | Volume of stock solution (µL) | Volume of methanol (µL) | Analyte concentration (ng/mL) |
| --- | --- | --- | --- | --- |
| CAL solution 1 | 200 | 10 | 990 | 2 |
| CAL solution 2 | 200 | 25 | 975 | 5 |
| CAL solution 3 | 200 | 100 | 900 | 20 |
| CAL solution 4 | 1000 | 50 | 950 | 50 |
| CAL solution 5 | 1000 | 200 | 800 | 200 |
| CAL solution 6 | 1000 | 1000 | 0 | 1000 |

The mass spectral conditions used to acquire data for target analytes and internal standards are reported in Supplement Table 2.

*Supplement Table 2.* *Mass spectral conditions for target analytes and internal standards.*

| **Analyte** | **Ionization mode** | **Retention time (min)** | **MRM** | **Declustering potential (V)** | **Collision energy (V)** | **Collision cell exit potential (V)** |
| --- | --- | --- | --- | --- | --- | --- |
| CBD | Positive | 6.16 | 315.1 → 193.1 | 71 | 27 | 16 |
|  |  |  | 315.1 → 123.0 | 71 | 41 | 8 |
| 7-OH-CBD | Negative | 2.67 | 329.1 → 299.1 | -75 | -20 | -15 |
|  |  |  | 329.1 → 268.1 | -75 | -34 | -17 |
| 7-COOH-CBD | Negative | 2.40 | 343.1 → 299.1 | -65 | -20 | -19 |
|  |  |  | 343.1 → 231.0 | -65 | -34 | -13 |
| Δ^9^-THC | Positive | 7.78 | 315.2 → 193.1 | 111 | 29 | 16 |
|  |  |  | 315.2 → 123.0 | 111 | 41 | 14 |
| 11-OH-Δ^9^-THC | Negative | 4.42 | 329.1 → 311.0 | -85 | -26 | -27 |
|  |  |  | 329.1 → 268.0 | -85 | -36 | -19 |
| 11-*nor*-9-COOH-Δ^9^-THC | Negative | 4.53 | 343.1 → 299.0  343.1 → 191.0 | -135  -135 | -28  -44 | -21  -11 |
| **Internal standard** | **Ionization mode** | **Retention time (min)** | **MRM** | **Declustering potential (V)** | **Collision energy (V)** | **Collision cell exit potential (V)** |
| CBD-D_3_ | Positive | 6.14 | 318.2 → 196.0 | 56 | 29 | 12 |
| 7-OH-CBD-D_3_ | Negative | 2.65 | 332.1 → 314.1 | -95 | -24 | -27 |
| 7-COOH-CBD-D_3_ | Negative | 2.38 | 346.1 → 302.1 | -40 | -20 | -21 |
| Δ^9^-THC-D_3_ | Positive | 7.76 | 318.2 → 196.1 | 126 | 31 | 14 |
| 11-OH-Δ^9^-THC-D_3_ | Negative | 4.40 | 332.2 → 314.2 | -90 | -28 | -19 |
| 11-*nor*-9-COOH-Δ^9^-THC-D_3_ | Negative | 4.51 | 346.1 → 302.1 | -110 | -28 | -21 |

### Method validation study design

A sensitive and specific liquid chromatograph tandem mass spectrometry (LC-MS^n^) method for determining CBD, 7-OH-CBD, 7-COOH-CBD, Δ^9^-THC, 11-OH-Δ^9^-THC, 11-*nor*-9-COOH-Δ^9^-THC in dog serum was validated according to the *M10 Bioanalytical Method Validation and Study Sample Analysis Guidance for Industry*.^^[[1]](#footnote-1)^^ The validation study investigated the specificity, limit of quantitation (LOQ), linearity and the linear range, accuracy, repeatability and intermediate precision, and dilution integrity.

*Linearity and linear range*

Six non-zero calibration samples (CALs) were prepared and analyzed in five independent runs to investigate linearity. Calibration curves were subjected to least-squares linear regression of the ratio of the areas of the quantifier ion for each analyte to that of the IS versus calibrator concentrations. Weighting was applied using 1/X where X was the analyte concentration in the respective calibrators. The linearity of the calibration line was assessed by examining the plot of residuals versus the fitted values and requiring that the mean value of the residuals was not different from zero.

The imprecision of the calculated concentration for each calibrator was required to be less than 20 % for LOQ and less than 15 % for all other calibrator concentrations.

#### Limit of quantification

The lower limit of quantification (LOQ) of each target analyte was defined as the concentration at which the quantifier and qualifier ion abundances for each analyte were characterized by a signal -to-noise ratio (*s/n*) greater than ten, the measured concentrations were within 20% of target concentrations, and the bias was less than 20%.

Three control samples containing each analyte at 0.2 ng/mL were independently prepared on each of three days. The concentrations of each analyte in these samples were determined and summary statistics for each were calculated. The precision, precision %CV, percent recovery, and percent bias at 0.200 ng/mL were calculated for each analyte.

#### Accuracy (recovery and bias) and precision (repeatability, within-batch, and intermediate precision)

The accuracy and precision of the LC-MS^n^ method for determining CBD, 7-OH-CBD, 7-COOH-CBD, Δ^9^-THC, 11-OH-Δ^9^-THC, 11-*nor*-9-COOH-Δ^9^-THC in dog serum were determined using control samples prepared at 2.00, 40.0, and 80.0 ng/mL in pooled dog serum. Three replicates of each of the control samples containing all analytes at three concentrations (*i.e*., LOW, MED, and HIGH) were analyzed in each of five batches for a total of fifteen determinations of each analyte at each concentration.

The accuracy was assessed for each analyte at each concentration by calculating the absolute recovery, the relative recovery, the absolute bias, and relative bias (expressed as a percentage). Absolute bias was calculated as the difference between the measured and the nominal concentrations, and the percentage bias was obtained by dividing the absolute bias by the nominal concentration and multiplying by one hundred. The 95% confidence interval for the percentage bias was calculated and compared to zero to determine significance of each percent bias estimate. If the relative bias was less than 15%, it was considered acceptable.

Precision was assessed by calculating the coefficient of variation (%CV) of precision estimates (*i.e*., repeatability, between-batch, and intermediate) based on measured concentrations of analytes in control materials. Precision was evaluated by analyzing three replicates of each of three control samples (*i.e*., LOW, MED, and HIGH) in each of five batches on separate days. The repeatability and between-batch precision were determined by one-way analysis of variance for each analyte at each concentration in five different batches. The intermediate precision was calculated as the square root of the sum of squares of the repeatability and between-batch precision estimates.

The % CV for the intermediate precision and the % bias were required to be less than 15 % except at concentrations less than the LOQ where they were required to be less than 20 %.

#### Selectivity, specificity, and carry over

The selectivity and specificity of the method were evaluated by analyzing drug-free dog serum samples from different sources to assess interference from endogenous substances. Also, the contribution of the IS to the response of each analyte was investigated by analyzing extracted blank serum supplemented only with the IS. The extent of carryover was estimated by analyzing extracted blank serum samples immediately after the analysis of the highest calibrator. The signals for interfering substances and carryover were required to be less than the LOQ.

#### Dilution effect

Dilution integrity was assessed by ten-fold dilution of serum samples supplemented with all analytes. These samples were analyzed in three replicates per batch over three batches (*i.e*., nine determinations of each analyte). Results were compared to the nominal concentrations. The relative bias and CV% of the intermediate precision were required to be less than 15 %.

#### Application

The validated LC-MS/MS method was successfully applied in the pharmacokinetic study of a full-spectrum cannabidiol hemp extract in MCT oil in beagle dogs. Blood samples were collected in evacuated serum tubes (red top) containing no anti-coagulants. After centrifugation at 3600 g for 5 min at 4◦C, the serum samples were transferred into propylene tubes and stored at − 80°C until they were analyzed by the method described in this study.

### Results and discussion

#### Linearity and linear range

Calibration ranges were 0.200-100 ng/mL for CBD, 7-OH-CBD, 7-COOH-CBD, Δ9-THC, 9-OH-D9-THC, and 11-*nor-*9-COOH-D9-THC. All calibration curves were determined using linear regression analysis with 1/X weighting.

Supplement Table 3. Results of linear regression analysis for calibration curves from the method validation study

| **Analyte** | **Batch** | **Slope** | **Intercept** | ***r*** | ***r*^2^** |
| --- | --- | --- | --- | --- | --- |
| CBD | 1 | 1.13010 | 0.00796 | 0.99999 | 0.99997 |
|  | 2 | 1.13016 | 0.01114 | 0.99995 | 0.99990 |
|  | 3 | 1.11677 | 0.01327 | 0.99999 | 0.99998 |
|  | 4 | 1.14600 | 0.00851 | 0.99999 | 0.99997 |
|  | 5 | 1.14445 | 0.00591 | 0.99999 | 0.99998 |
| 7-OH-CBD | 1 | 0.75384 | -0.00015 | 0.99999 | 0.99998 |
|  | 2 | 0.72846 | -0.00045 | 0.99998 | 0.99997 |
|  | 3 | 0.73355 | 3.41E-05 | 0.99992 | 0.99985 |
|  | 4 | 0.75443 | 0.000153 | 0.99997 | 0.99994 |
|  | 5 | 0.75992 | -0.00016 | 0.99996 | 0.99993 |
| 7-COOH-CBD | 1 | 1.03565 | 0.00466 | 1.000000 | 1.000000 |
|  | 2 | 1.01808 | 0.00412 | 0.99999 | 0.99999 |
|  | 3 | 1.00907 | 0.00422 | 0.99996 | 0.99992 |
|  | 4 | 1.02980 | 0.00415 | 0.99999 | 0.99998 |
|  | 5 | 102454 | 0.00241 | 0.99999 | 0.99998 |
| Δ^9^-THC | 1 | 1.02926 | 0.00918 | 0.99998 | 0.99996 |
|  | 2 | 1.03957 | 0.02394 | 0.99994 | 0.99989 |
|  | 3 | 0.99749 | 0.00131 | 0.99983 | 0.99965 |
|  | 4 | 1.05162 | 0.02480 | 0.99995 | 0.99991 |
|  | 5 | 1.05021 | 0.02428 | 0.99998 | 0.99996 |
| 11-OH-Δ^9^-THC | 1 | 0.97102 | 0.00267 | 0.99998 | 0.99996 |
|  | 2 | 0.99818 | 0.00172 | 0.99998 | 0.99997 |
|  | 3 | 0.93597 | 7.58E-05 | 0.99985 | 0.99997 |
|  | 4 | 0.96624 | 0.00309 | 0.99999 | 0.99999 |
|  | 5 | 1.00156 | 0.00105 | 0.99996 | 0.99992 |
| 11-*nor*-9-COOH-Δ^9^-THC | 1 | 1.06509 | -0.00181 | 0.99991 | 0.99982 |
|  | 2 | 1.16315 | 0.00759 | 0.99976 | 0.99951 |
|  | 3 | 1.06892 | 0.00828 | 0.99995 | 0.99990 |
|  | 4 | 1.13612 | 0.00334 | 0.99995 | 0.99991 |
|  | 5 | 0.98207 | 0.00295 | 0.99993 | 0.99986 |

Calibration curves were linear across the concentration range 0.200-100 ng/mL for each analyte. Coefficients of determination (*r*^2^) of the weighted linear regression equations were 0.999 or greater (Supplement Table 3). Plots of residuals versus fitted concentration were randomly distributed and the mean values of the residuals were not different from zero.

#### Limit of Quantification

The LOQ for each analyte was 0.2 ng/mL of Greyhound dog serum. The intermediate precision % CV at 0.2 ng/mL was less than 20% (Supplemental Table 4) and % bias at 0.2 ng/mL was less than 20%. Specifically, the intermediate precision % CV was 14.4% for D^9^-THC (Supplemental Table 4) and the % bias was -3.73% for 11-*nor-*9-COOH-D^9^-THC (), respectively.

#### Accuracy and precision

The results of accuracy and precision are reported in Supplemental Table 5. Mean intra- and inter-day accuracies were between − 13.7 % and 14.7 % for all the analytes. Intra- and inter-day precision CVs were less than 14.3 %. Overall, the results for precision and accuracy met the validation criteria for these measurements.

#### Selectivity, specificity, cross talk and carry over

For the selectivity and specificity analysis, endogenous substances did not interfere with the analytes or IS (interferences were less than the LOQ of each analyte). Additionally, there was no cross-talk effect between the analytes and IS (less than 2.0 % of CAL1 for all analytes) and no significant carry over effect was detected for all analytes and IS (less than 6.0 % of CAL1 for all the molecules).

#### Dilution effects

The relative bias and CV% of the intermediate precision are reported in Supplemental Table 4. All results for dilution effects met the requirements for relative bias and CV% of the intermediate precision being less than 15 %. less than 15 %.

#### Application

To our knowledge, this method is one of a few validated LC-MS/MS methods for quantifying serum concentrations of CBD and its metabolites, 7-OH-CBD and 7-COOH-CBD, and Δ^9^-THC and its metabolites, 11-OH-Δ^9^-THC, and 11-*nor*-9-COOH-Δ^9^-THC in dogs. A validated LC-MS/MS method for determining CBD, 7-OH-CBD, 7-COOH-CBD, Δ^9^-THC, 11-OH-Δ^9^-THC, 11-*nor*-9-COOH-Δ^9^-THC, CBDA, THCA, cannabigerol, cannabinol, and 11-*nor*-9-carboxy-Δ^9^-THC glucuronide was recently reported by Wakshlag *et al*. [1]. This method was reported to have somewhat higher lower limits of quantification than those of the method reported in the present study. Furthermore, a validated LC-MS/MS method for determining CBD and Δ^9^-THC plus major Phase 1 oxidative metabolites in dog samples from a study of the pharmacokinetics 1:20 THC:CBD herbal extract in dogs by Chicoine *et al*. [2] and subsequently used to investigate the pharmacokinetics of a similar herbal product in cats [3].

Our method has been successfully applied in the analysis of serum samples from studies of the disposition of CBD and Δ^9^-THC and two of their oxidative metabolites after IV and PO administration of CBD dominant hemp products to beagle dogs.

Supplemental Table 4. LOQ, Linearity, and Dilution Integrity

| **Parameter** | **Analytes** | | | | | |
| --- | --- | --- | --- | --- | --- | --- |
|  | **CBD** | **7-OH-CBD** | **7-COOH-CBD** | **Δ^9^-THC** | **11-OH-Δ^9^-THC** | **11-*nor*-9-COOH-Δ^9^-THC** |
| **Standard curve, ng/mL)** | 0.2-100 | 0.2-100 | 0.2-100 | 0.2-100 | 0.2-100 | 0.2-100 |
| **Linear Range, ng/mL** | 0.2-100 | 0.2-100 | 0.2-100 | 0.2-100 | 0.2-100 | 0.2-100 |
| **Coefficient of determination, *r*^2^** | 0.99999 | 0.99992 | 0.99996 | 0.99983 | 0.99985 | 0.99995 |
| **LOQ (ng/mL)** | 0.20 | 0.20 | 0.20 | 0.20 | 0.20 | 0.20 |
| **%CV at LOQ** | 7.08 | 3.92 | 8.82 | 14.4 | 12.0 | 12.9 |
| **% Bias at LOQ** | -2.53 | -1.87 | -3.72 | 0.0190 | 0.375 | -3.73 |
| **% Recovery at LOQ** | 97.5 | 98.1 | 96.3 | 100 | 100.4 | 96.3 |
| **Dilution Integrity, 10X %CV (n=9)** | 1.25 | 1.77 | 1.26 | 1.90 | 2.84 | 3.19 |
| **Dilution Integrity 10X, %Recovery (n=9)** | 97.5 | 98.1 | 96.3 | 100 | 100 | 96.3 |

Supplemental Table 5. Precision and Accuracy Estimates from QC Data

| **Parameter** | **Analytes** | | | | | |
| --- | --- | --- | --- | --- | --- | --- |
|  | **CBD** | **7-OH-CBD** | **7-COOH-CBD** | **Δ^9^-THC** | **11-OH-Δ^9^-THC** | **11-*nor*-9-COOH-Δ^9^-THC** |
| **Repeatability (n=15)** | - | - | - | - | - | - |
| **LQC** | 3.27% | 2.05% | 1.42% | 1.84% | 2.05% | 1.42% |
| **MQC** | 1.16% | 0.674% | 1.42% | 0.478% | 1.66% | 1.88% |
| **HQC** | 3.27% | 2.05% | 1.42% | 1.84% | 2.05% | 1.42% |
| **Inter-day precision (n=15)** |  |  |  |  |  |  |
| **LQC** | 0.565% | 1.06% | 6.59% | 5.21% | 6.63% | 6.59% |
| **MQC** | 2.48% | 4.96% | 7.05% | 5.69% | 6.01% | 7.05% |
| **HQC** | 1.90% | 5.25% | 5.10% | 5.31% | 5.02% | 6.35% |
| **Intermediate precision (n=15)** |  |  |  |  |  |  |
| **LQC** | 3.31% | 5.22% | 6.74% | 5.52% | 6.94% | 6.74% |
| **MQC** | 2.73% | 5.00% | 7.30% | 5.71% | 6.23% | 7.30% |
| **HQC** | 2.16% | 5.32% | 5.14% | 5.34% | 5.35% | 6.47% |
| **Recovery (n=15)** |  |  |  |  |  |  |
| **LQC** | 98.8% | 95.4% | 92.9% | 96.8% | 98.3% | 108% |
| **MQC** | 99.3% | 95.5% | 93.9% | 96.3% | 98.7% | 107% |
| **HQC** | 99.6% | 92.2% | 93.0% | 95.9% | 95.7% | 105% |
| **Bias (n=15)** |  |  |  |  |  |  |
| **LQC** | -1.17% | -4.60% | -7.07% | -3.16% | -1.66% | 7.90% |
| **MQC** | -0.718% | -4.53% | -6.06% | -3.71% | -1.29% | 6.65% |
| **HQC** | -1.52% | -7.80% | -6.99% | -4.12% | -4.33% | 4.63% |

LQC=2.0 ng/mL, MQC=20.0 ng/mL; HQC=80 ng/mL

**REFERENCES**

[1] Wakshlag, J.J., Schwark, W.S., Deabold, K.A., Talsma, B.N., Cital, S., Lyubimov, A., Iqbal, A. and Zakharov, A. (2020) Pharmacokinetics of Cannabidiol, Cannabidiolic Acid, Delta9-Tetrahydrocannabinol, Tetrahydrocannabinolic Acid and Related Metabolites in Canine Serum After Dosing With Three Oral Forms of Hemp Extract. *Front Vet Sci* **7**, 505.

[2] Chicoine, A., Illing, K., Vuong, S., Pinto, K.R., Alcorn, J. and Cosford, K. (2020) Pharmacokinetic and Safety Evaluation of Various Oral Doses of a Novel 1:20 THC:CBD Cannabis Herbal Extract in Dogs. *Front Vet Sci* **7**, 583404.

[3] Lyons, C., McEwan, K., Munn-Patterson, M., Vuong, S., Alcorn, J. and Chicoine, A. (2024) Pharmacokinetic of two oral doses of a 1:20 THC:CBD cannabis herbal extract in cats. *Front Vet Sci* **11**, 1352495.

1. <https://www.fda.gov/media/162903/download> [↑](#footnote-ref-1)
